# Integrative genomic and functional analyses identify LjbZIP36 as a candidate regulator of β-carotene accumulation during floral color transition in *Lonicera japonica*

**DOI:** 10.1186/s12870-026-09757-3

**Published:** 2026-08-14

**Authors:** Meiyu Qiao, Xiaoyu Su, Lei Li, Lina Wang, Yiwen Cao, Yongliang Yu, Chunming Li, Dandan Lu, Yao Sun, Mengfan Su, Zhengwei Tan, Huizhen Liang

**Affiliations:** 1 Institute of Chinese Herbal Medicines, Henan Academy of Agricultural Sciences, Zhengzhou, 450002 China; 2 Key Laboratory for the Protection and Utilization of Chinese Herbal Medicine Resources of Henan Province, Zhengzhou, Henan 450002 China

**Keywords:** *Lonicera japonica*, bZIP transcription factor, Genome-wide analysis, Carotenoid biosynthesis, Flower color transition

## Abstract

**Background:**

Floral color transition is a characteristic developmental process in *Lonicera japonica*, during which flowers gradually change from white to golden yellow during flower maturation owing to carotenoid accumulation. Although carotenoid biosynthetic genes have been identified in *L. japonica*, the transcriptional regulatory mechanisms underlying carotenoid accumulation during floral color transition remain poorly understood. Basic leucine zipper (bZIP) transcription factors are important regulators of plant growth, development, and secondary metabolism; however, their potential involvement in carotenoid biosynthesis in *L. japonica* has not been systematically investigated.

**Results:**

Using a recently released chromosome-level reference genome, we performed a comprehensive genome-wide analysis of the bZIP transcription factor family in *L. japonica* and identified 68 *LjbZIP* genes, which were classified into 13 conserved subfamilies. Integrating phylogenetic analysis, expression profiling across floral developmental stages, and transcriptome–metabolome association analyses, we identified LjbZIP36 as a candidate regulator associated with carotenoid accumulation during floral color transition. The expression of *LjbZIP36* increased markedly during late floral development and exhibited a positive correlation with β-carotene accumulation. Subcellular localization analysis demonstrated that LjbZIP36 is a nucleus-localized protein. Transient overexpression of *LjbZIP36* in *Nicotiana benthamiana* promoted carotenoid accumulation, particularly β-carotene, and enhanced the expression of several carotenoid biosynthetic genes. Furthermore, yeast one-hybrid and dual-luciferase assays indicated that LjbZIP36 can interact with and activate the promoters of *LjPSY*, *LjPDS*, *LjZDS*, and *LjLCYB*, key genes involved in carotenoid biosynthesis.

**Conclusions:**

This study provides the genome-wide characterization of the bZIP transcription factor family in *L. japonica* and identifies LjbZIP36 as a candidate positive regulator associated with carotenoid accumulation during floral color transition. These findings expand our understanding of the transcriptional regulation of carotenoid metabolism in honeysuckle and provide valuable genetic resources for future functional studies and molecular breeding of ornamental and medicinal cultivars.

**Supplementary Information:**

The online version contains supplementary material available at https://doi.org/10.1186/s12870-026-09757-3.

## Introduction

*Lonicera japonica* Thunb. (honeysuckle) is one of the most important medicinal plants in East Asia and has been widely used in traditional Chinese medicine for centuries because of its anti-inflammatory, antiviral, antioxidant, and antibacterial and other properties [1–6]. Its dried flower buds, known as “Jinyinhua”, are officially recorded in the Chinese Pharmacopoeia and are extensively utilized in both pharmaceutical and healthcare industries [6]. In addition to its medicinal importance, *L. japonica* possesses considerable ornamental value owing to its characteristic floral color transition during flower development. The flowers gradually change from white to golden yellow as blooming progresses, forming a visually distinctive developmental process that is closely associated with changes in pigment metabolism [7, 8]. This natural color transition provides an attractive model for investigating the molecular mechanisms underlying flower pigmentation and secondary metabolite accumulation in medicinal plants.

Flower coloration in plants is primarily determined by the accumulation of betalains, anthocyanins, carotenoids, and chlorophylls [9]. Previous studies have demonstrated that the white-to-yellow transition of *L. japonica* flowers is mainly attributable to carotenoid accumulation rather than anthocyanin biosynthesis [8]. Among the carotenoids detected in honeysuckle flowers, β-carotene represents one of the most abundant components and shows substantial accumulation during late floral development, coinciding with the appearance of yellow pigmentation [7]. Beyond their role as pigments, carotenoids are essential tetraterpenoid metabolites that participate in photosynthesis, photoprotection, photomorphogenesis, and environmental adaptation [10, 11]. They also serve as precursors of important signaling molecules and phytohormones, including abscisic acid (ABA) and strigolactones (SLs) [12, 13], thereby influencing plant growth and development [14, 15]. Furthermore, carotenoids possess significant nutritional and pharmaceutical value because of their antioxidant properties and provitamin A activity [16–18]. Therefore, elucidating the regulatory mechanisms controlling carotenoid accumulation during floral color transition is of both biological and practical significance.

The carotenoid biosynthetic pathway has been extensively characterized in higher plants. Starting from isoprenoid precursors generated through the methylerythritol phosphate pathway, carotenoids are synthesized through a series of enzymatic reactions catalyzed by key structural genes, including geranylgeranyl diphosphate synthase (*GGPPS*), phytoene synthase (*PSY*), phytoene desaturase (*PDS*), ζ-carotene desaturase (*ZDS*), and lycopene cyclases (Fig. S1) [19, 20]. Among these enzymes, phytoene synthase (PSY) is generally regarded as the major rate-limiting enzyme of the pathway [21–23], whereas lycopene β-cyclase (LCYB) plays a critical role in directing metabolic flux toward β-carotene biosynthesis [24, 25]. Increasing evidence indicates that carotenoid accumulation is regulated not only by structural genes but also by complex transcriptional regulatory networks. Although several carotenoid biosynthetic genes have been identified in *L. japonica* [7, 8, 26], the upstream transcription factors responsible for coordinating their expression during floral color transition remain largely unknown.

Transcription factors are central regulators of plant secondary metabolism because they control the expression of biosynthetic genes through specific interactions with promoter cis-elements. In recent years, multiple transcription factor families, including MYB, bHLH, NAC, WRKY, ERF, and bZIP proteins, have been reported to participate in carotenoid biosynthesis and accumulation (Fig. S1) [20, 27–29]. Among them, basic leucine zipper (bZIP) transcription factors constitute one of the largest and most evolutionarily conserved transcription factor families in plants. Typical bZIP proteins contain a highly conserved basic DNA-binding region and a leucine zipper dimerization domain, enabling them to regulate downstream gene expression through sequence-specific DNA binding [30, 31]. Beyond their well-established roles in plant development, hormone signaling, and stress responses, several bZIP transcription factors have recently been implicated in carotenoid metabolism [32–37]. For example, AtHY5 directly activates the expression of *PSY* in Arabidopsis in response to light signals [38], whereas SlHY5 promotes carotenoid accumulation in tomato fruits through transcriptional activation of carotenoid biosynthetic genes (*SlPSY1* and *SlPDS* genes) [39]. In mango, MibZIP66 enhances β-carotene accumulation by targeting the promoter of *MiPSY* [34], while CsbZIP44 positively regulates carotenoid biosynthesis in citrus through activation of multiple pathway genes [40]. These findings collectively suggest that bZIP transcription factors represent important regulators of carotenoid metabolism; however, their potential involvement in floral carotenoid accumulation remains poorly understood in medicinal plants.

With the recent release of a chromosome-level reference genome for *L. japonica*, it has become possible to systematically investigate transcription factor families and their regulatory functions at the whole-genome scale. Although previous studies have identified carotenoid metabolic pathways and several transcriptional regulators associated with floral coloration in *L. japonica*, the regulatory network remains incomplete. In particular, whether members of the bZIP transcription factor family participate in carotenoid biosynthesis has not yet been investigated.

In the present study, we performed a genome-wide identification and characterization of the bZIP transcription factor family in *L. japonica* using the latest chromosome-scale genome assembly. A total of 68 *LjbZIP* genes were identified and systematically analyzed with respect to their chromosomal distribution, phylogenetic relationships, gene structures, conserved motifs, and expression profiles during floral development. By bioinformatic characterization, phylogenetic clustering, and integrating transcriptomic and metabolomic datasets, we identified LjbZIP36 as a candidate regulator strongly associated with β-carotene accumulation. Functional analyses, including subcellular localization, transient overexpression, yeast one-hybrid, and dual-luciferase assays, further suggested that LjbZIP36 can activate the expression of multiple carotenoid biosynthetic genes. Our findings provide new insights into the transcriptional regulation of carotenoid metabolism during floral color transition and establish a valuable genomic resource for future molecular breeding and functional studies in *L. japonica*.

## Materials and methods

### Plant materials and growth conditions

Five-year-old *Lonicera japonica* cultivar ‘Miyinhua 1’ (GFLJ) was cultivated in the resource nursery of the Institute of Chinese Herbal Medicines, Henan Academy of Agricultural Sciences. Flower buds and blooms at five developmental stages (S1–S5, Fig. 4A) were separately harvested for metabolome and transcriptome analyses. For each stage, floral materials were pooled from no fewer than five individual plants to serve as one biological replicate, with three independent biological replicates prepared for all analyses. All samples were immediately frozen in liquid nitrogen and stored at − 80 °C until further use.

### Genome-wide identification and sequence analysis of the *bZIP* genes from *L. japonica*

The genome sequences of *L. japonica* were obtained from the Genome Database [41]. A total of 161 known bZIP family protein sequences, including 70 from *A. thaliana* and 89 from *Oryza sativa*, were retrieved to construct a Hidden Markov Model (HMM) using HMMER 3.0. The HMM profile was used to search the *L. japonica* proteome to identify putative bZIP proteins. In parallel, BLASTP (ncbi-blast-v2.10.1+) [42] using an E-value cutoff of e^− 20^ to identify additional homologous sequences. Candidate sequences obtained from the HMM and BLASTP searches were merged and further validated using PfamScan (v1.6) against the Pfam-A database (v33.1) [43, 44]. Only proteins containing both PF07716 and PF00170 domains were retained as final bZIP members. A total of 68 *LjbZIP* genes were identified.

Physicochemical properties, including amino acid length, molecular weight, and isoelectric point, were predicted using ProtParam (ExPASy) [45]. Subcellular localization was predicted using WoLF PSORT and Plant-mPLoc.

### Phylogenetic analysis of *bZIP* genes

Full-length bZIP protein sequences from *A. thaliana* were downloaded from the TAIR database (http://www.arabidopsis.org/) (Table S2). Multiple sequence alignment was performed using MAFFT v7.427. A neighbor-joining (NJ) phylogenetic tree was constructed in MEGA10 using the p-distance model, with partial deletion (site coverage cutoff: 50%) and 1,000 bootstrap replicates. The phylogenetic tree was visualized using iTOL v6 (https://itol.embl.de/).

### Analysis of conserved motifs, gene structure, and domains

To better characterize the sequence similarity and diversity of conserved motifs among LjbZIP proteins in honeysuckle, conserved motifs of LjbZIP proteins were identified using MEME suite v5.0.5 (http://meme-suite.org/), with the maximum number of motifs set to 0. Gene structure (exon–intron organization) and domain distribution were visualized using TBtools v1.130 [46] based on genome annotation and sequence data.

### Prediction of cis-regulatory elements in promoters

The 2,000 bp upstream sequences from the transcription start site of each *LjbZIP* gene were extracted from the *L. japonica* genome. Promoter sequences were analyzed using PlantCARE (http://bioinformatics.psb.ugent.be/webtools/plantcare/html/) to identify putative cis-regulatory elements.

### Chromosome localization and collinearity analysis

Genomic coordinates of *LjbZIP* genes were retrieved from genome annotation files and mapped onto chromosomes using MG2C (http://mg2c.iask.in/mg2c_v2.1/). Collinearity analysis was performed using MCScanX following all-vs-all BLASTP (E-value < 1e^− 5^). Collinear blocks were identified with default parameters (MATCH_SCORE: 50; MATCH_SIZE: 5; GAP_PENALTY: -1; OVERLAP_WINDOW: 5; E_VALUE: 1e^− 05^; MAX GAPS: 25).

### Transcriptome-based expression profiling and candidate gene screening

To identify candidate *bZIP* genes potentially involved in carotenoid accumulation during floral color transition, transcriptome datasets from five floral developmental stages were analyzed. RNA-seq datasets from five floral developmental stages were used to analyze expression patterns of *LjbZIP* genes. Gene expression levels were quantified as TPM (Transcripts Per Million) with log2 transformation across stages. Differential gene expression analysis was performed using DESeq2, with thresholds set as: adjusted p-value (FDR) < 0.05, |log2 fold change| ≥ 1, All analyses were based on three biological replicates per stage.

To identify candidate *bZIP* genes potentially involved in carotenoid accumulation during floral color transition, genes were prioritized based on the following criteria: significant differential expression during floral development; expression patterns positively correlated with carotenoid accumulation, particularly β-carotene content; phylogenetic clustering with previously reported carotenoid-associated bZIP transcription factors from other plant species.

Based on these criteria, candidate genes were selected for further analysis. Quantitative real-time PCR (qRT-PCR) was performed to validate expression patterns. Total RNA was extracted using the FastPure^®^ Universal Plant Total RNA Isolation Kit (Vazyme, China). First-strand cDNA was synthesized using HiScript^®^ II Q RT SuperMix (+ gDNA wiper). qRT-PCR was performed on an ABI 7500 Fast Real-Time PCR system using SYBR Green Master Mix (GenStar, China). The thermal cycling conditions were: 95 °C for 2 min, followed by 40 cycles of 95 °C for 10 s and 60 °C for 30 s. Melt curve analysis was performed to confirm amplification specificity. Relative expression levels were calculated using the 2^−ΔΔCt^ method with G6PD as the internal reference gene.

### Bioinformatic characterization and subcellular localization of LjbZIP36

Multiple sequence alignment of LjbZIP36 and homologous proteins (Table S4) from other plant species was performed using DNAMAN v6.0.3.99 (LynnonBiosoft). Subcellular localization was predicted using WoLF PSORT and experimentally validated via transient expression of *LjbZIP36–EGFP* fusion constructs in *Nicotiana benthamiana* leaves. Fluorescence signals were observed using a Leica DM2000 confocal laser scanning microscope (Leica Microsystems, USA) at 48 h post-infiltration.

### Transient overexpression of *LjbZIP36* in *Nicotiana benthamiana* leaves

The full-length coding sequence of *LjbZIP36* was cloned into the pCAMBIA3300B vector. Recombinant plasmids were introduced into *Agrobacterium tumefaciens* strain GV3101. Bacterial suspensions were prepared in infiltration buffer (10 mM MES, 10 mM MgCl₂, 200 µM acetosyringone, pH 5.6). Leaves of 35-day-old *N. benthamiana* plants were infiltrated with Agrobacterium carrying either *LjbZIP36* constructs or empty vector control.

Samples were collected 6 days post-infiltration for further analysis. The tobacco actin gene was used as an internal reference for qRT-PCR. Expression of carotenoid biosynthetic genes (*PSY*, *PDS*, *ZDS*, and *LCYB*) was analyzed following the same qRT-PCR procedure described above. The primer sequences for these genes are listed in Table S3.

### Determination of carotenoid accumulation in transiently transformed *N. benthamiana* leaves

Leaf segments from infiltrated areas were harvested and weighed (fresh weight, g). Total carotenoids were extracted using acetone ethanol (1:1, v/v) at room temperature for 48 h. Absorbance was measured at multiple wavelengths (665, 642, 485, and 474 nm), and carotenoid content was calculated using established equations based on chlorophyll correction.

β-carotene and (E/Z)-phytoene contents were detected by MetWare (Wuhan, China) based on the AB Sciex QTRAP 6500 + LC-MS/MS platform. Briefly, fresh floral tissues were freeze-dried, ground into powder, and extracted with a mixed solvent of n-hexane, acetone, and ethanol (1:1:1, v/v/v). The extracts were analyzed using UPLC-APCI-MS/MS with scheduled multiple reaction monitoring (MRM). Carotenoid compounds were identified and quantified using authentic standards and MultiQuant software. Method validation parameters are summarized in Table S8. β-carotene contents were calculated based on freeze-dried weight and expressed as µg. g^− 1^ DW. For quality control, a pooled QC sample prepared by mixing equal volumes of representative samples was analyzed periodically throughout the LC-MS/MS sequence. The stability of the analytical system was evaluated by overlapping total ion chromatograms (TICs) of QC samples.

### Yeast one-hybrid assay

Based on transcriptome analysis and correlation with carotenoid accumulation, four key carotenoid biosynthetic genes (*LjPSY*, *LjPDS*, *LjZDS* and *LjLCYB*) were selected for interaction analysis. Promoter fragments of these genes were cloned into the pLacZi vector, while *LjbZIP36* CDS was inserted into the pB42AD vector. Recombinant constructs were co-transformed into yeast strain EGY48. Empty pB42AD vector served as the negative control.

Transformants were screened on SD selective media. Positive colonies were serially diluted and spotted onto selective plates containing X-Gal to assess potential DNA–protein interactions based on growth and blue coloration.

### Dual-luciferase reporter assay

Approximately 2,000 bp promoter regions of target genes were cloned into the pGreenII 0800-LUC vector to generate reporter constructs. *LjbZIP36* CDS was cloned into the pCAMBIA3300B vector as the effector. Constructs were co-infiltrated into *N. benthamiana* leaves together with pSoup-p19.

After 48 h, luciferase activity was measured using a Dual-Luciferase Reporter Assay System (Promega, USA). Luminescence was detected using a SpectraMax iD3 microplate reader (Molecular Devices, USA). LUC/REN ratios were calculated to evaluate the transcriptional activation ability of LjbZIP36. Four biological replicates were performed for each assay.

### Statistical analysis

All experiments were conducted with at least three biological replicates. Data are presented as mean ± standard deviation (SD). Statistical analyses were performed using SPSS 26.0 software (IBM Corp., Armonk, NY, USA). Significant differences between two groups were evaluated using Student’s t-test, whereas multiple comparisons were analyzed by one-way analysis of variance (ANOVA) followed by Duncan’s multiple range test. Differences were considered statistically significant at *P* < 0.05 or *P* < 0.01. For RNA-seq data, false discovery rate (FDR) correction was applied to control for multiple testing.

## Results

### Genome-wide identification and characterization of *bZIP* genes in *L. japonica*

**Fig. 1:**
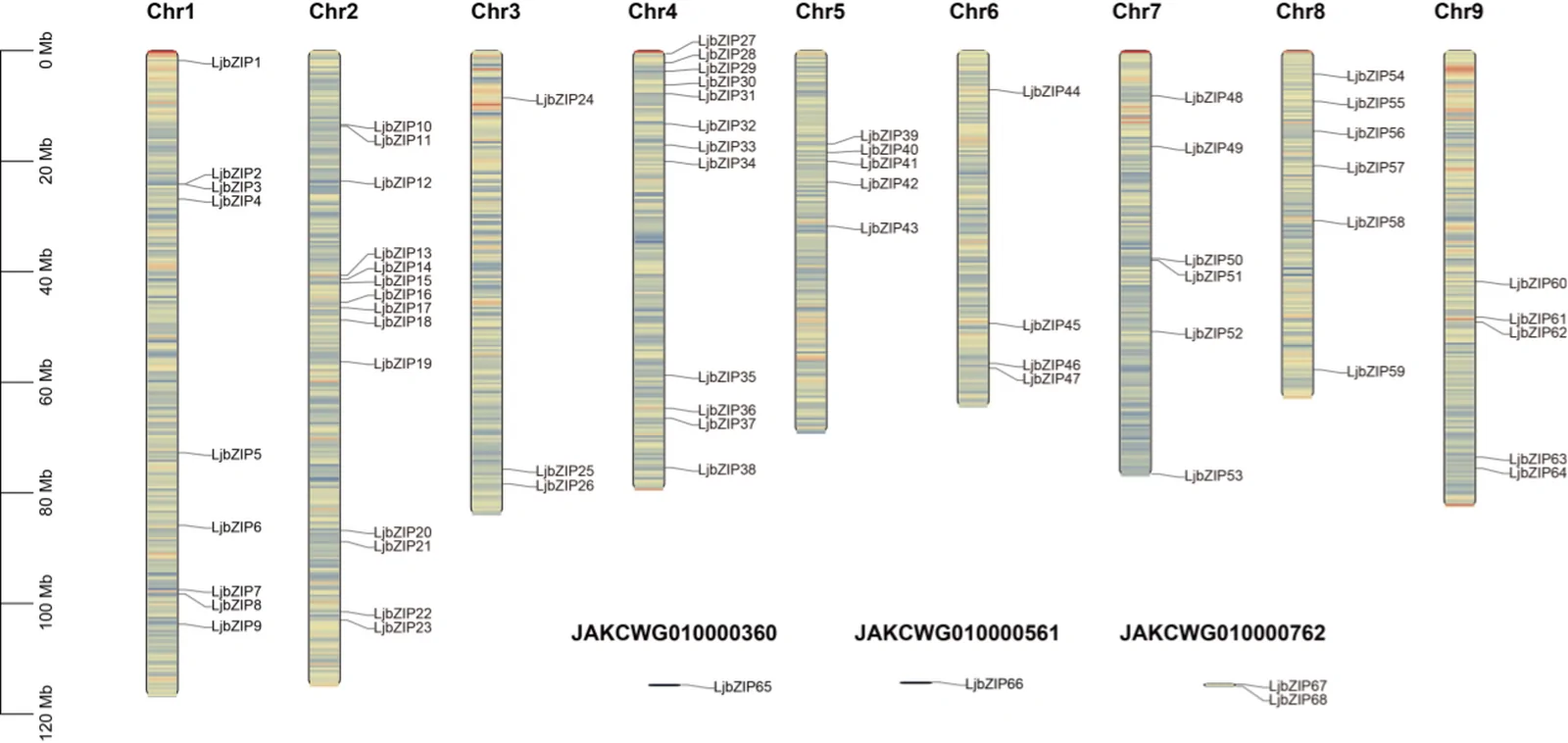
Chromosomal distribution of *LjbZIP* family genes. Chr1–Chr9 correspond to the nine chromosomes of honeysuckle, with the left-side scale marking chromosome physical length. The blue curve shows gene density distribution, while black lines denote the exact chromosomal loci of *LjbZIP* genes

Genome-wide identification of transcription factor families is a widely used approach to systematically explore regulatory components in plant species. A total of 68 *bZIP* transcription factor genes were identified in the *L. japonica* genome after removing redundant and incomplete sequences based on HMMER and BLASTp searches combined with Pfam domain confirmation. These genes were designated *LjbZIP1* to *LjbZIP68* according to their chromosomal positions (Fig. 1). The *LjbZIP* genes were unevenly distributed across the nine chromosomes of *L. japonica*, with an additional four genes located on unanchored scaffolds. Gene density varied significantly among chromosomes, with longer chromosomes generally harboring more *LjbZIP* members. Notably, gene clusters were observed in several chromosomal regions, including the upper region of chromosome 4 and the central region of chromosome 2, suggesting local duplication events may have contributed to gene family expansion (Fig. 1).

Physicochemical property analysis showed that LjbZIP proteins ranged from 117 to 777 amino acids in length, with molecular weights of 13.97–83.75 kDa, isoelectric points of 4.50–10.24, and instability indices of 31.73–88.75 (Table S1). Subcellular localization prediction consistently indicated nuclear localization for all LjbZIP proteins, supporting their conserved role as transcription factors.

**Fig. 2:**
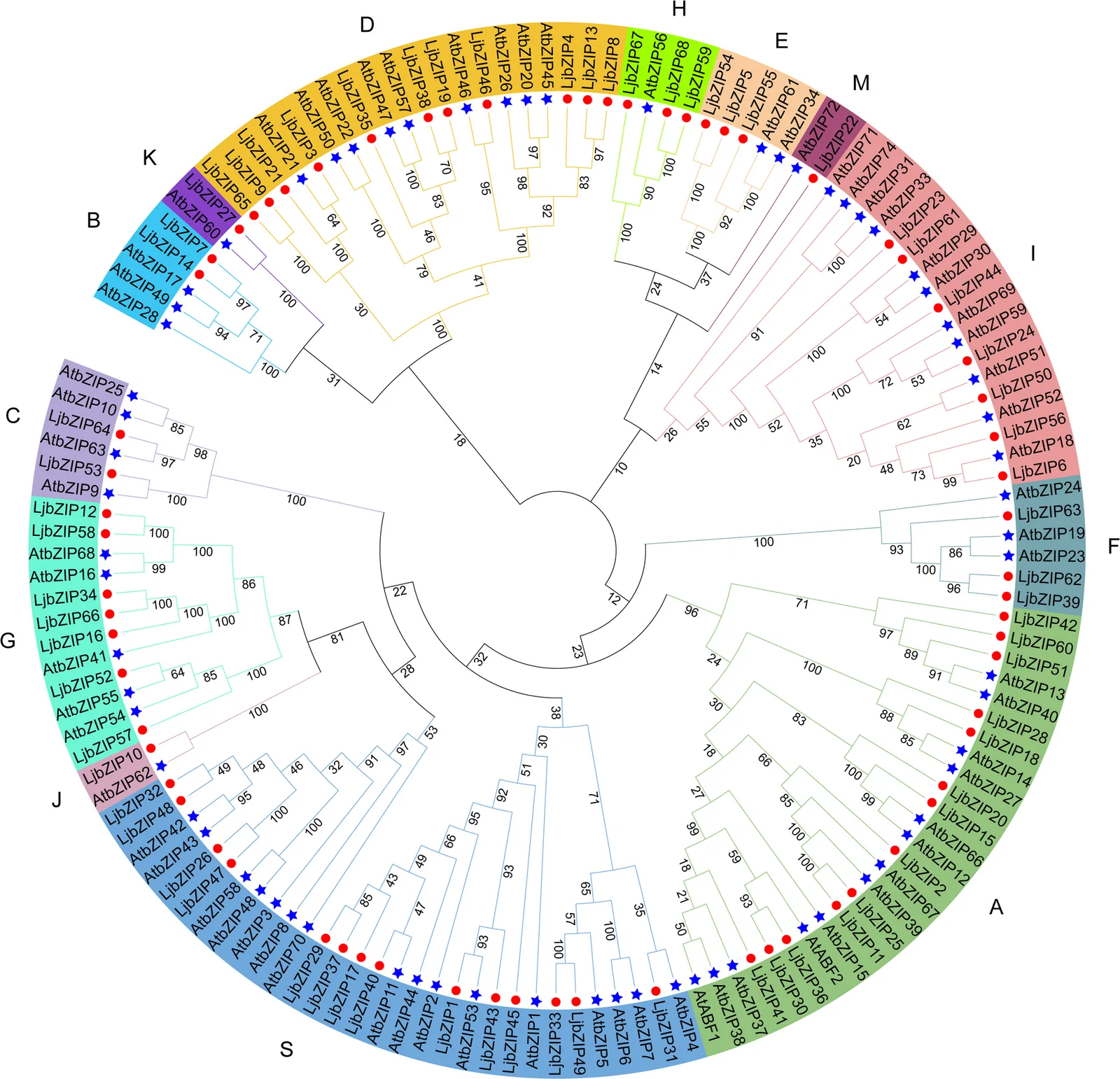
Phylogenetic analysis of bZIP proteins. The unrooted phylogenetic tree classified these proteins into 13 distinct subfamilies, which are distinguished by different color labeling. Proteins derived from honeysuckle and *A. thaliana* are labeled with red circles and blue asterisks, respectively. The phylogenetic tree was constructed based on the neighbor-joining method, with 1000 bootstrap replicates

Phylogenetic analysis classified the LjbZIP family into 13 subfamilies (A–M), each containing members from both *L. japonica* and *Arabidopsis thaliana* (Fig. 2). The S subfamily was the largest, whereas the M and K subfamilies contained the fewest members. The presence of all major bZIP clades suggests that the family diversification occurred prior to the divergence of these species and is highly conserved in angiosperms.

### Gene structure and conserved motif analysis of *LjbZIP* family

**Fig. 3:**
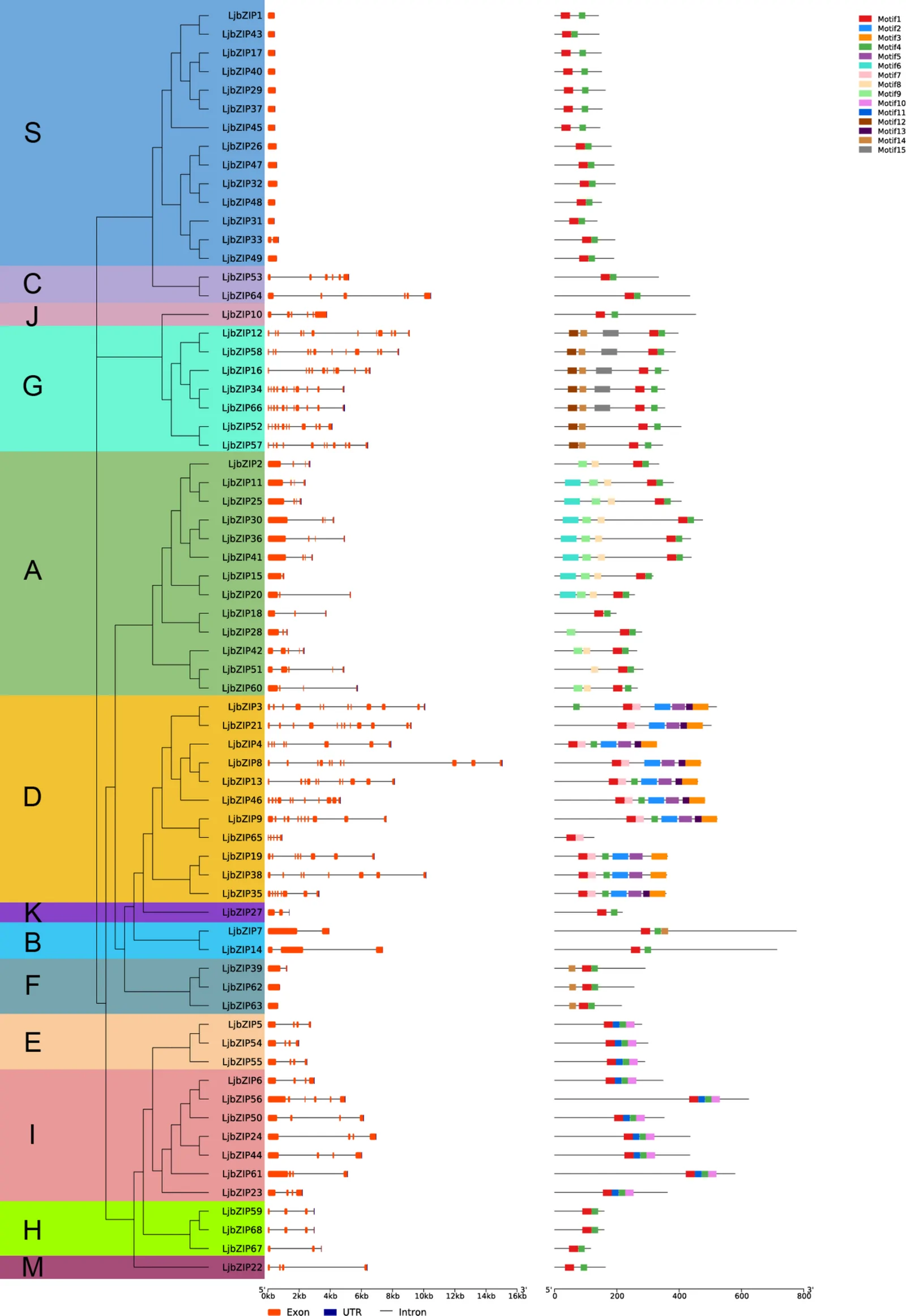
Phylogenetic relationships, gene structures and motif compositions of *L. japonica* LjbZIP proteins

To investigate structural conservation, phylogenetic relationships were integrated with gene structure and conserved motif analyses. Members within the same subfamily exhibited similar exon–intron organization, whereas clear structural divergence was observed among different clades. Subfamily S genes generally contained fewer exons, while subfamily D members displayed more complex gene structures (Fig. 3).

A total of 15 conserved motifs were identified across LjbZIP proteins. All members contained the core bZIP signature motif (Motif 1) (Fig. 3), indicating strong conservation of functional domains. Subfamily-specific motif compositions were also observed (Fig. 3), suggesting functional diversification among clades.

Sequence logo analysis further confirmed the conservation of the bZIP domain. In the basic region, the canonical N-X7-R/K motif was strictly conserved, including invariant asparagine (Asn, N) and arginine (Arg, R) (Fig. S2). In the leucine zipper region, periodic hydrophobic leucine residues were conserved, maintaining the structural basis for dimerization (Fig. S2).

### Promoter cis-element analysis and chromosomal collinearity

Promoter analysis identified multiple cis-acting regulatory elements in *LjbZIP* genes, including light-responsive (G-box, Box4), hormone-responsive (ABRE, ERE), and stress-related elements (ARE, STRE) (Fig. S3). The widespread presence of light- and hormone-responsive elements suggests that *LjbZIP* genes may be tightly regulated by environmental and hormonal signals.

Collinearity analysis revealed extensive syntenic relationships between *LjbZIP* genes and their homologs in both *Arabidopsis thaliana* and *Oryza sativa* (Fig. S4). More collinear gene pairs were identified with *A. thaliana* (Fig. S4A and B), reflecting their closer evolutionary relationship. Intraspecific synteny analysis identified 21 high-confidence duplicated gene pairs (Fig. S4C), indicating that segmental and whole-genome duplication events have played major roles in *LjbZIP* family expansion in *L. japonica*. All duplicated *LjbZIP* genes were further classified into three groups based on the size of their homologous clusters (Fig. S4D). The classification of duplicated *LjbZIP*s was fully consistent with the intraspecific synteny results, clearly resolving the hierarchical homologous relationships among duplicated *LjbZIP* copies. Collectively, these synteny analyses revealed the evolutionary footprint of genomic duplication that drove *LjbZIP* family expansion in honeysuckle, laying a foundation for subsequent functional divergence analysis of duplicated *LjbZIP* genes.

### Expression profiling of *LjbZIP* genes during floral development

**Fig. 4:**
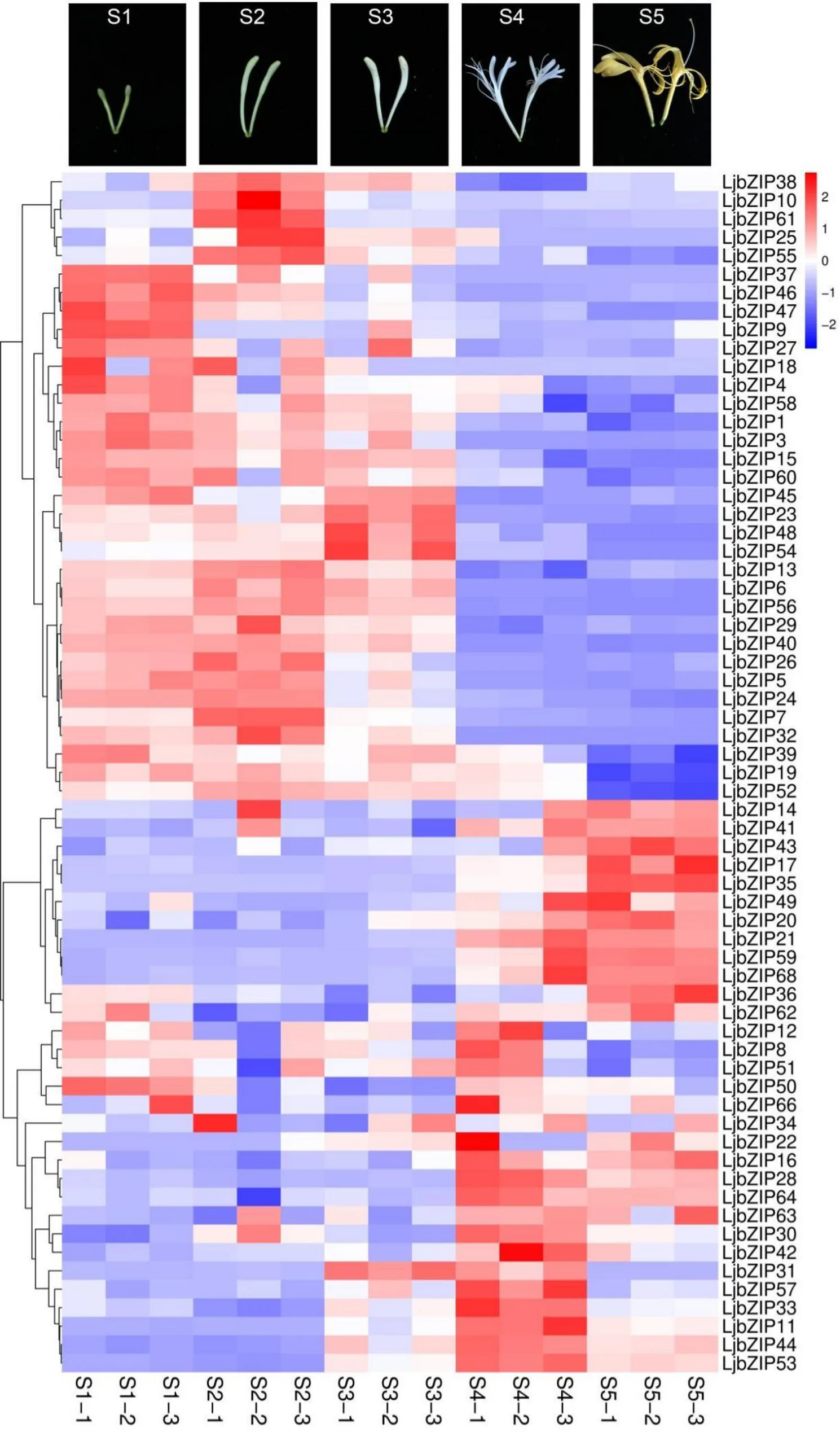
Expression profiling heat map of *LjbZIP* genes across floral developmental stages of *L. japonica*. S1, green buds. S2, slightly white buds. S3, whole white buds. S4, silver flowers (initial blooming, white flowers). S5, golden flowers (fully opened, yellow flowers)

Transcriptome analysis across five floral developmental stages (S1–S5) revealed distinct temporal expression patterns of *LjbZIP* genes (Fig. 4). Several genes showed high expression in early stages (S1–S3), while others were preferentially expressed in late stages (S4–S5) coinciding with the onset of floral color transition. Among them, *LjbZIP36* displayed a marked stage-specific expression pattern, with transcript levels significantly increasing at S4 and reaching a peak at S5, consistent with the white-to-yellow pigmentation shift.

In addition, *LjbZIP36* showed a positive association with β-carotene accumulation across developmental stages, suggesting a potential involvement in carotenoid biosynthesis during floral color transition. Compared with other *LjbZIP* members, *LjbZIP36* displayed a more trait-consistent temporal expression pattern during the critical color-transition window (Table S6). Based on its coordinated late-stage expression dynamics, metabolite association, and supporting phylogenetic relationship with previously characterized carotenoid-related bZIP transcription factors, LjbZIP36 was selected for further functional characterization.

### Identification and characterization of LjbZIP36

**Fig. 5:**
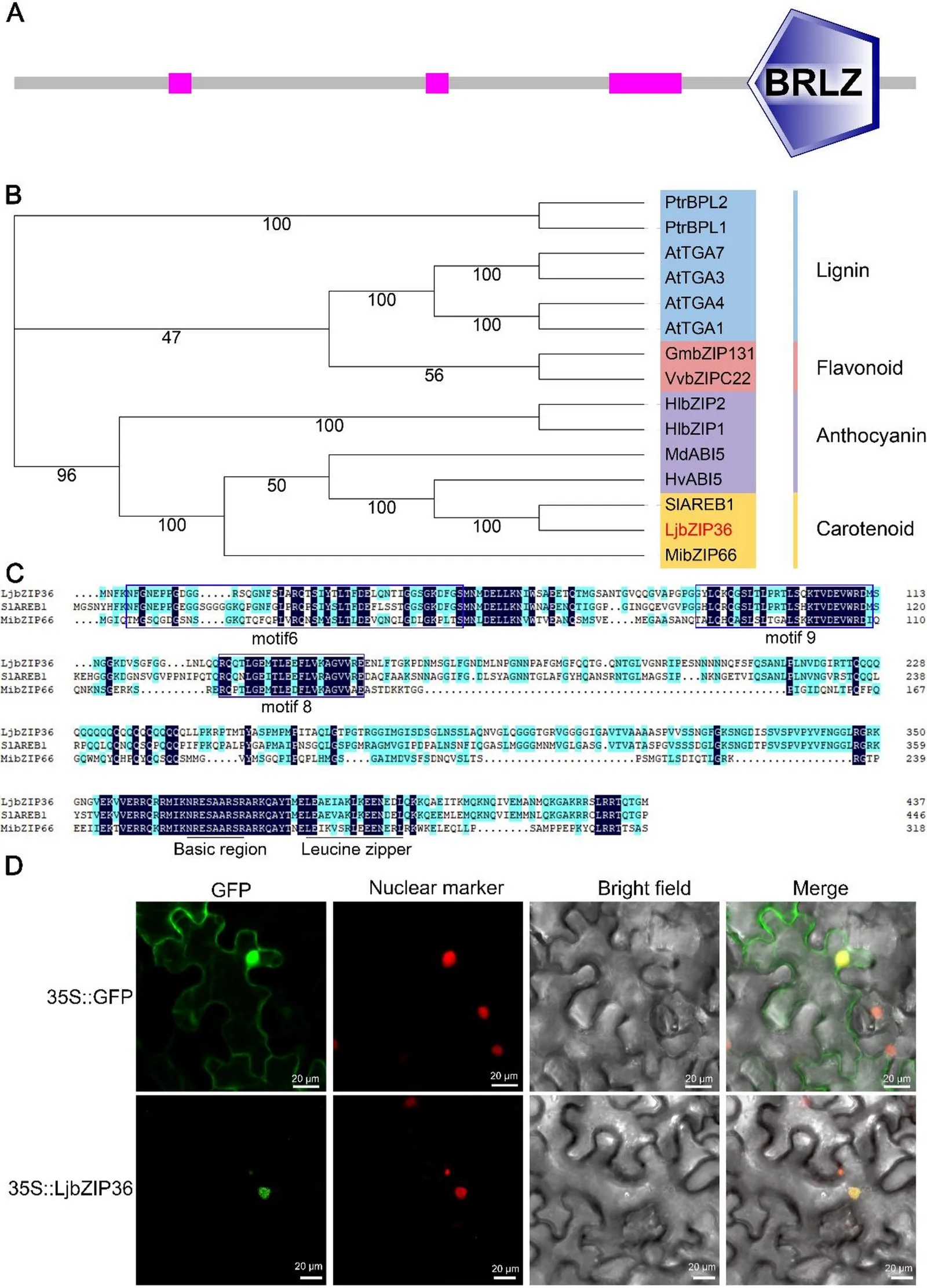
Bioinformatic characterization and subcellular localization of the candidate carotenoid regulator LjbZIP36. **A** Conserved domain architecture of LjbZIP36, as predicted by the SMART database (http://smart.embl-heidelberg.de/). LjbZIP36 contains a canonical basic region leucine zipper (BRLZ) domain at the C-terminus. Pink boxes represent low-complexity regions, and the blue hexagon indicates the conserved BRLZ domain, the defining structural feature typical of bZIP transcription factors. **B** Phylogenetic analysis of LjbZIP36 with functionally characterized bZIP proteins from other plant species that regulate different secondary metabolic pathways. LjbZIP36 (marked in red) clusters with the carotenoid biosynthesis regulators SlAREB1 from *Solanum lycopersicum* and MibZIP66 from *Malus domestica*. Bootstrap values are shown at each branch node. Colored bars on the right indicate the secondary metabolic pathway associated with each bZIP. The protein sequences used to construct the phylogenetic tree are listed in Table S4. **C** Conserved motif alignment of LjbZIP36 with SlAREB1 and MibZIP66. The conserved core motifs 6, 8, and 9 are labeled, and the canonical basic region and leucine zipper structure characteristic of bZIP proteins are highlighted. **D** Subcellular localization of LjbZIP36 in *Nicotiana benthamiana* leaf epidermal cells. 35 S::GFP (empty vector) was used as a control, which showed GFP fluorescence distributed throughout the cell. The 35 S::LjbZIP36-GFP fusion protein co-localized with the nuclear marker (red fluorescence), confirming nuclear localization. Scale bars = 20 μm

Domain prediction confirmed that LjbZIP36 contains a complete BRLZ domain characteristic of functional bZIP transcription factors (Fig. 5A). Phylogenetic analysis showed that LjbZIP36 clustered with known carotenoid-related bZIP regulators, including SlAREB1 and MibZIP66 (Fig. 5B). Sequence alignment further confirmed conservation of key functional domains required for DNA binding and dimerization (Fig. 5C). Subcellular localization assays in *Nicotiana benthamiana* demonstrated that LjbZIP36-GFP fluorescence was exclusively localized in the nucleus, consistent with its predicted role as a transcription factor (Fig. 5D).

### *LjbZIP36* expression correlates positively with β-carotene accumulation

**Fig. 6:**
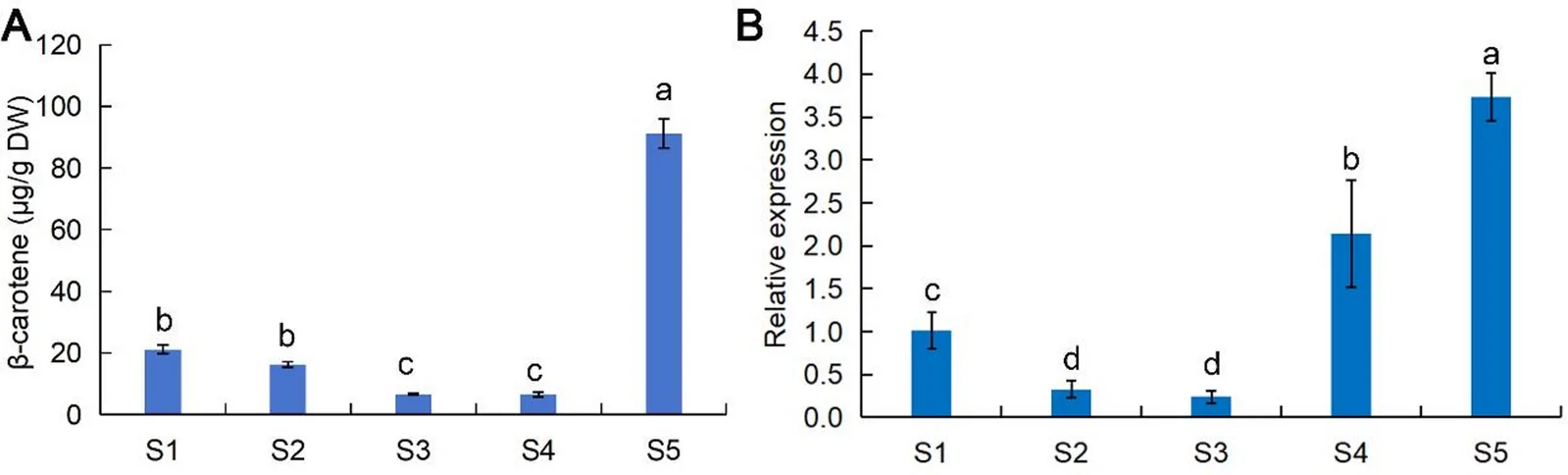
Correlation between *LjbZIP36* expression and β-carotene accumulation during floral development of *Lonicera japonica*. **A** Changes in β-carotene content across five floral developmental stages (S1–S5). Data are presented as mean ± SD (*n* = 3). Different lowercase letters on the bars indicate significant differences (Duncan’s multiple range test, F (4, 10) = 383.98, *P* < 0.0001). **B** Relative expression levels of *LjbZIP36* during floral development determined by qRT-PCR. Data are presented as mean ± SD (*n* = 3). Different lowercase letters on the bars indicate significant differences (Duncan’s multiple range test, F (4, 10) = 23.39, *P* < 0.001)

Metabolomic profiling across floral developmental stages revealed that β-carotene was the dominant carotenoid component and showed a strong accumulation trend during the white-to-yellow transition. Integrated transcriptome–metabolome analysis indicated that LjbZIP36 expression showed a positive association with β-carotene accumulation across floral developmental stages (Fig. 6A and B; Fig. S5; Fig. S6; Table S6). Consistently, *LjbZIP36* expression was significantly upregulated at the S5 stage compared with S4 (Fig. 4). qRT-PCR validation confirmed that *LjbZIP36* expression closely followed β-carotene accumulation across developmental stages (Fig. S6;Fig. 6B), supporting its potential involvement in carotenoid biosynthesis regulation.

### LjbZIP36 promotes carotenoid biosynthesis in *Nicotiana benthamiana*

**Fig. 7:**
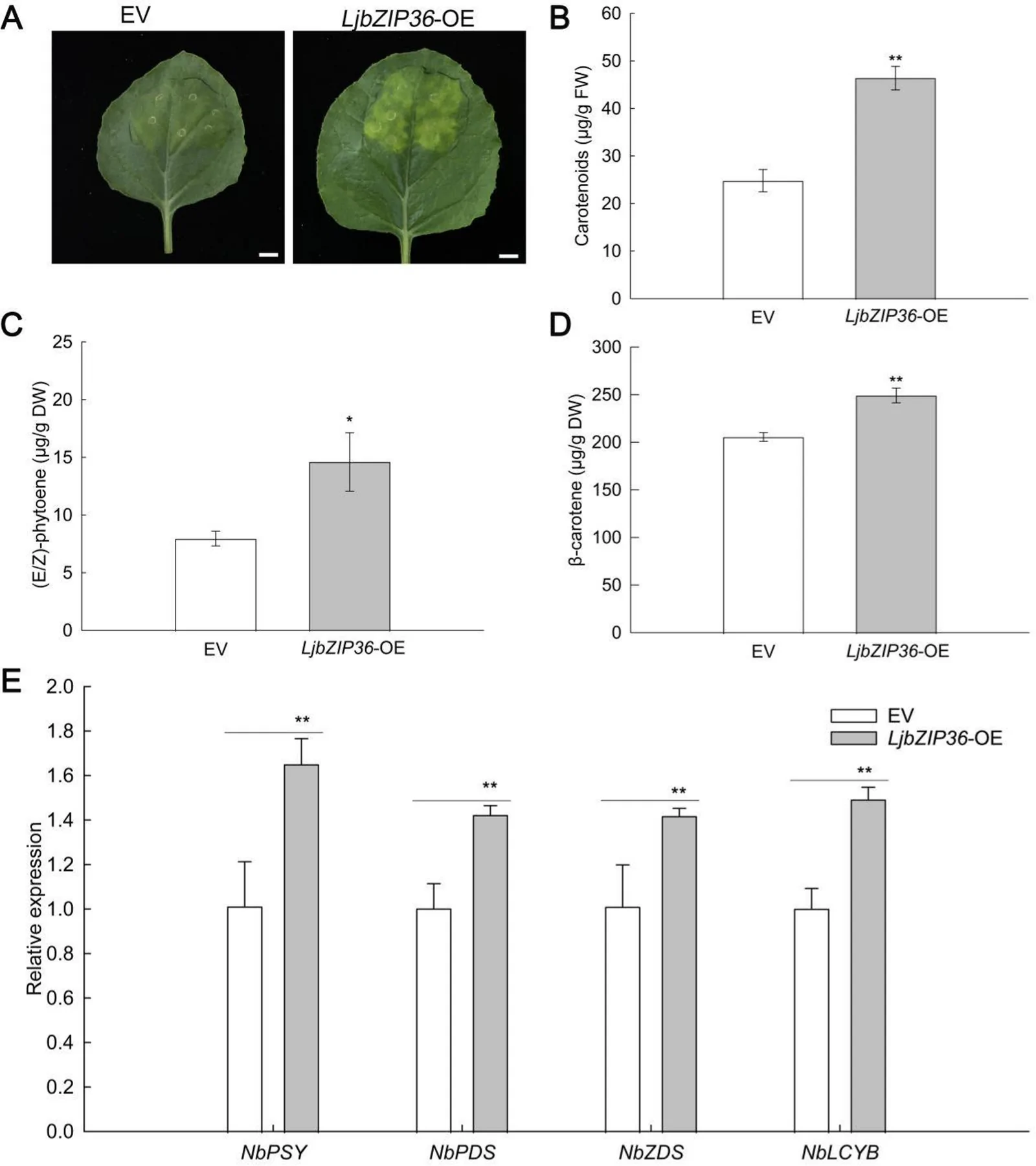
Effect of LjbZIP36 on carotenoid accumulation. **A** Phenotypic changes in *N. benthamiana* leaves after transient overexpression of *LjbZIP36* (6 days after Agrobacterium injection). Scale bars: 1 cm. **B** Analysis of total carotenoid content in leaves overexpressing *LjbZIP36*. Significant differences, as determined by Student’s t-test, are indicated by asterisks (**P < 0.01). **C **and** D** Changes in the contents of major carotenoid components in leaves overexpressing *LjbZIP36*. Significant differences, as determined by Student’s t-test, are indicated by asterisks (*P < 0.05, **P < 0.01). **E** Relative expression of carotenoid biosynthesis structural genes in *LjbZIP36*-overexpressing vs. EV-control in *N. benthamiana*. Statistical significance was determined by two-tailed Student’s t-test with Bonferroni correction for multiple comparisons. The significance threshold was adjusted to α = 0.0125 (0.05/4). **, *P* < 0.0125, data are presented as mean ± SD (*n* = 3)

*Nicotiana benthamiana* transient expression system was employed as a heterologous system to preliminarily investigate the function of LjbZIP36. Following Agrobacterium-mediated infiltration, leaves expressing *LjbZIP36* displayed a visible yellowing phenotype compared with the empty vector control (Fig. 7A). Although chlorophyll contents were not quantified in the present study, LC–MS/MS analysis demonstrated a significant increase in absolute β-carotene content, suggesting that carotenoid accumulation is likely the major contributor to the observed phenotype. Quantitative biochemical analysis showed that total carotenoid content was significantly increased in *LjbZIP36*-overexpressing leaves (Fig. 7B). LC-MS/MS profiling further revealed that β-carotene exhibited the most pronounced increase among the detected carotenoid components (Fig. 7D). Consistently, several endogenous carotenoid biosynthetic genes, including *NbPSY*,* NbPDS*, *NbZDS*, and *NbLCYB*, were significantly upregulated in *LjbZIP36*-expressing tissues (Fig. 7E).

### LjbZIP36 activates carotenoid biosynthetic genes

**Fig. 8:**
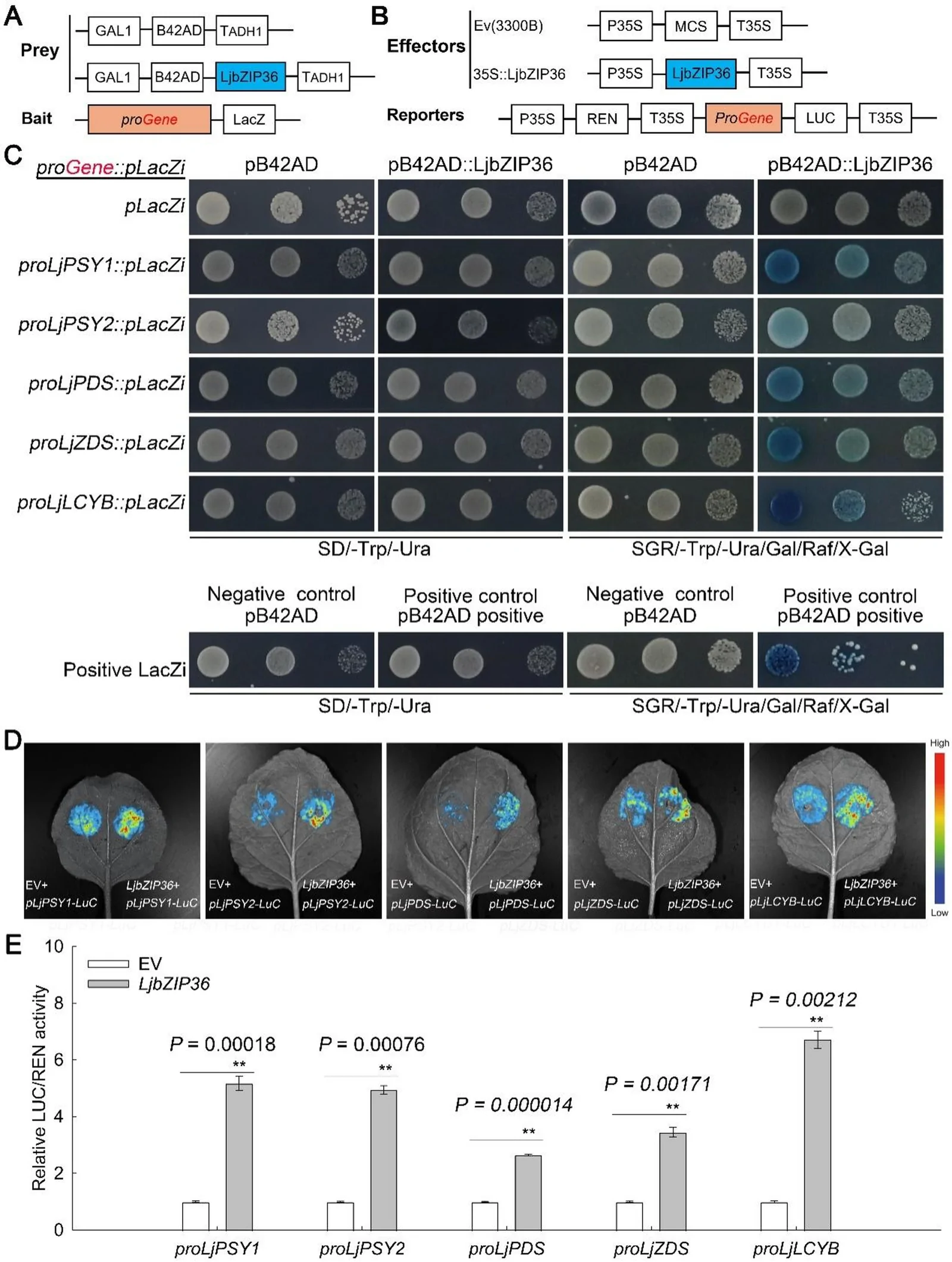
Interaction and transcriptional activity analysis of LjbZIP36 with promoters of key genes involved in carotenoid biosynthesis. **A** Schematic diagram of vector construction used in yeast one-hybrid assay. **B** Schematic diagram of vector construction for dual-luciferase reporter system. The reporter vector contains the promoters of *LjPSY*, *LjPDS*, *LjZDS* or *LjLCYB* fused to firefly luciferase (LUC). **C** Yeast one-hybrid results reveal the interaction between LjbZIP36 and the corresponding promoters. **D** LUC imaging analysis of the effect of LjbZIP36 on the activities of target gene promoters. **E** Dual-luciferase assay results. EV represents the empty vector control. Statistical significance was determined by two-tailed Student’s t-test with Bonferroni correction for five independent comparisons. The significance threshold was adjusted to α = 0.01 (0.05/5). Exact adjusted *P*-values are shown above each column, **P < 0.01 after correction. Data are presented as mean ± SD (*n* = 4)

Combined with transcriptome analysis of the expression patterns of key genes in the carotenoid biosynthesis pathway, we found that the expression levels of *PSY*, *ZDS*, *PDS*, and *LCYB*, a key enzyme gene in the β‑carotene branch, were upregulated at the late stage of flower development. Yeast one-hybrid assays demonstrated that LjbZIP36 binds to the promoters of *LjPSY*, *LjPDS*, *LjZDS*, and *LjLCYB* (Fig. 8A and C). Dual-luciferase assays further confirmed that LjbZIP36 significantly enhanced the transcriptional activity of these promoters (Fig. 8B, D and E). Among them, *LjLCYB* showed the strongest activation response, suggesting that LjbZIP36 may preferentially regulate the β-carotene branch of the carotenoid biosynthetic pathway.

## Discussion

### Expansion and evolutionary conservation

The basic leucine zipper (bZIP) transcription factor family is one of the most conserved regulatory systems in land plants, with established roles in development, stress responses, and secondary metabolism [36, 37, 47–51]. In this study, 68 *LjbZIP* genes were identified from the chromosome-level genome of *L. japonica*, providing a genomic framework for functional characterization of this family in honeysuckle (Fig. 1; Table S1). The size of the LjbZIP family is comparable to that reported in other dicot species such as *Arabidopsis thaliana* [52] and tomato [53], suggesting a conserved evolutionary scale of this gene family in angiosperms. In contrast, species such as soybean exhibit expanded bZIP repertoires due to additional whole-genome duplication events [54], indicating that lineage-specific polyploidy rather than functional necessity largely determines family size variation.

Phylogenetic analysis grouped LjbZIP proteins into 13 subfamilies (Fig. 2), consistent with the conserved classification of plant bZIPs [55]. Structural analysis confirmed the presence of canonical BRLZ domains across all members (Fig. 3), supporting their conserved transcription factor identity. Collinearity analysis indicated that segmental duplication was the major driver of *LjbZIP* expansion (Fig. S4), suggesting that whole-genome duplication events contributed to regulatory diversification in *L. japonica*. This pattern is consistent with the evolutionary expansion mechanisms of many plant transcription factor families [56–60] and reflects the importance of large-scale genomic events in shaping regulatory complexity in perennial species.

Importantly, this genome-wide identification provides a necessary foundation for screening functional candidates involved in secondary metabolism, rather than serving as a purely descriptive gene family analysis.

### LjbZIP36 acts as a candidate pathway-level regulator of carotenoid biosynthesis

Floral color transition in *L. japonica* is primarily driven by carotenoid accumulation during late developmental stages [7]. To identify potential regulatory genes, we integrated phylogenetic information, developmental transcriptomics, and metabolite–gene correlation analysis. Among the identified *LjbZIP* family members, *LjbZIP36* was prioritized because it exhibited coordinated late-stage expression during the floral color transition, a positive association with β-carotene accumulation, and phylogenetic proximity to previously characterized carotenoid-related bZIP transcription factors (Figs. 4 and 6; Fig. S5; Fig. S6; Table S6). Functional assays demonstrated that LjbZIP36 is a nuclear-localized transcription factor (Fig. 5D) capable of activating multiple key carotenoid biosynthetic genes, including *LjPSY*, Lj*PDS*, *LjZDS*, and *LjLCYB* (Fig. 8). Notably, PSY represents the rate-limiting entry point of carotenoid biosynthesis [21–23], while LCYB controls metabolic flux toward β-carotene formation [24, 25]. These genes represent both upstream rate-limiting (PSY) and flux-controlling branch-point enzymes (LCYB), indicating that LjbZIP36 likely acts at a pathway coordination level rather than regulating a single enzymatic step. It is important to note that transcriptional regulation of carotenoid biosynthesis in *L. japonica* is likely a multi-factorial process. Previous studies have demonstrated that LjMADS36 positively regulates *LjLCYB* during floral coloration [61]. Because *LjLCYB* represents a common downstream target identified in both studies, it is conceivable that multiple transcription factor families coordinately regulate its expression. Promoter sequence analysis indicates that *LjLCYB* contains several putative cis-regulatory elements, including canonical G-box/ABRE motifs recognized by bZIP proteins and CArG-box motifs typically bound by MADS-box transcription factors. Therefore, *LjbZIP36* and *LjMADS36* may function through combinatorial rather than mutually exclusive regulatory mechanisms. Future studies integrating promoter deletion analysis, ChIP-seq and transcription factor co-expression assays will be valuable for testing this hypothesis. Therefore, LjbZIP36 may not function as a solitary master regulator but rather represent an additional layer of transcriptional control contributing to regulatory redundancy or combinatorial regulation in carotenoid biosynthesis. Transient expression in *Nicotiana benthamiana* further supported its positive regulatory role, as *LjbZIP36* overexpression increased carotenoid biosynthesis gene expression and enhanced β-carotene accumulation (Fig. 7), consistent with its proposed regulatory function.

Collectively, these findings suggest that LjbZIP36 contributes to the transcriptional regulation of carotenoid biosynthesis, expanding the functional repertoire of bZIP transcription factors in floral pigmentation regulation.

### Regulatory model, limitations, and future perspectives

Based on our results, we propose that LjbZIP36 functions as a transcriptional activator coordinating multiple carotenoid biosynthetic genes during late floral development, thereby contributing to β-carotene accumulation and floral color transition in *L. japonica*.

However, several aspects of the regulatory mechanism remain unresolved. First, the specific cis-regulatory elements recognized by LjbZIP36 have not been experimentally defined, and whether it binds canonical bZIP motifs such as G-box or ABRE elements remains to be determined. Second, carotenoid biosynthesis is regulated by multiple transcription factor families, including MADS-box and MYB proteins; therefore, potential functional redundancy or cooperative interactions between LjbZIP36 and other regulators such as LjMADS36 cannot be excluded. Third, although transient expression assays provide preliminary evidence supporting its regulatory role, more robust in vivo mechanistic validation is still required.

In this study, we used yeast one-hybrid and dual-luciferase reporter assays as complementary approaches to provide initial evidence for transcriptional activation of target promoters by LjbZIP36 in vitro and in planta. Although EMSA and transient loss-of-function approaches are technically feasible, they were beyond the scope of the present study because of time and resource constraints. By contrast, stable genetic transformation and genome-wide ChIP analyses in *Lonicera japonica* remain technically challenging.

Future studies employing ChIP-seq or CUT&RUN approaches will be essential to define the genome-wide binding landscape of LjbZIP36 and construct a comprehensive transcriptional regulatory network governing carotenoid metabolism.

## Conclusions

This study provides a comprehensive genome-wide characterization of the LjbZIP transcription factor family in *Lonicera japonica*, expanding the current understanding of this important gene family in honeysuckle. Through integrative bioinformatic and functional analyses, we identified LjbZIP36 as a previously uncharacterized member that functions as a nucleus-localized transcriptional activator. LjbZIP36 activates key genes in the carotenoid biosynthetic pathway, including *LjPSY*, *LjPDS*, *LjZDS*, and *LjLCYB*, thereby promoting carotenoid accumulation during the floral color transition from white to yellow.

Collectively, our findings identify *LjbZIP36* as a candidate transcriptional regulator contributing to carotenoid biosynthesis in *L. japonica*, providing mechanistic insight into floral pigmentation. This study also identifies LjbZIP36 as a promising molecular target for genetic improvement of honeysuckle cultivars with enhanced ornamental traits and potential medicinal value.

## Supplementary Information

Supplementary Material 1: Fig. S1. Carotenoid metabolic pathways and their regulation in plants and algae. Fig. S2. The calculated consensus logos of the highly conserved basic region and leucine zipper region across all LjbZIP proteins in honeysuckle. The bit values represent the information content of each amino acid at a specific position in a sequence. Fig. S3. Cis-acting element distribution in *LjbZIP* promoters. Grid color and number reflect the copy number of distinct cis-acting elements. Fig. S4. *bZIP* genes co-linearity analysis. (A) Co-linearity analysis between honeysuckle and *Arabidopsis thaliana*. (B) Co-linearity analysis between honeysuckle and *Oryza sativa*. (C) Intraspecies co-linearity analysis of honeysuckle. Gene names/family names are labeled on the outermost layer. Colored circles represent different chromosomes. Gray lines indicate whole-genome collinearity within the species, and colored lines represent the collinearity of this gene family within the species. (D) Classification of duplicated *LjbZIP*s. Light blue, green, and yellow respectively represent homologous relationships among 2, 3, and 4 members of *LjbZIP*s. Fig. S5 Correlation analysis between gene expression profiles and carotenoid metabolites based on integrated transcriptomic and metabolomic datasets across five floral developmental stages (S1-S5). S1, green buds. S2, slightly white buds. S3, whole white buds. S4, silver flowers (initial blooming, white flowers). S5, golden flowers (fully opened, yellow flowers). Rows represent genes and columns represent metabolites. Red and blue indicate positive and negative correlations, respectively. (A) S1 vs S2. (B) S1 vs S3. (C) S1 vs S4. (D) S1 vs S5. (E) S2 vs S3. (F) S2 vs S4. (G) S2 vs S5. (H) S3 vs S4. (I) S3 vs S5. (J) S4 vs S5. Fig. S6 Relationship between *LjbZIP36* expression and β-carotene accumulation during floral development of *Lonicera japonica* flowers from S1 to S5. Fig. S7 Representative LC–MS/MS chromatograms used for carotenoid identification and quantification. Chromatograms are shown for blank, authentic standard, empty vector (EV), and *LjbZIP36*-overexpressing (*LjbZIP36-OE*) samples. (A) β-carotene. (B) (E/Z)-phytoene.

Supplementary Material 2: Table S1. Characteristics of identified *LjbZIP* genes in *Lonicera japonica*. Table S2. bZIP transcription factors from *Arabidopsis thaliana* used for phylogenetic analysis. Table S3. Primer sequences used in this study. Table S4. bZIP proteins used for phylogenetic and comparative analyses of LjbZIP36. Table S5. Expression levels (FPKM values) of *LjbZIP* genes across floral developmental stages. Table S6. Comprehensive screening information of *LjbZIP* family members associated with β-carotene accumulation during floral development (Pearson's |r| ≥ 0.8, *p*-value ＜ 0.05). Table S7. Data of quantitative real-time PCR. Table S8 Method validation parameters for carotenoid quantification by LC-MS/MS.

## Data Availability

The raw sequence data presented in this paper have been deposited in the Genome Sequence Archive (GSA) (Chen et al., 2021) (https://ngdc.cncb.ac.cn/gsa) of the National Genomics Data Center (NGDC) (Members and Partners, 2025), a joint initiative of the China National Center for Bioinformation and the Beijing Institute of Genomics, Chinese Academy of Sciences, under the accession number CRA027573.

## Abbreviations

bZIP: basic leucine zipper
Asn/N: Asparagine
Arg/R: Arginine
Lys/K: Lysine
ABA: Abscisic acid
SLs: Strigolactones
PSY: Phytoene synthase
PDS: Phytoene desaturase
ZDS: Zeta-carotene desaturase
LCYB: Lycopene β-cyclase
LCYE: Lycopene ε-cyclase
*L. japonica*: *Lonicera japonica*
*A. thaliana*: *Arabidopsis thaliana*
*N. benthamiana*: *Nicotiana benthamiana*
CDS: Coding Sequence
PCR: Polymerase chain reaction
qRT-PCR: quantitative Real-Time PCR
EV: Empty vector
GFP: Green fluorescent protein
ABRE: ABA-responsive element
ARE: Antioxidant response elements
STRE: Stress response element
ERE: Ethylene responsive element
