# Supplementary material for: Integrative genomic and functional analyses identify LjbZIP36 as a candidate regulator of β-carotene accumulation during floral color transition in Lonicera japonica
Source: BMC Plant Biol. 2026 Aug 14;26:1926. doi: 10.1186/s12870-026-09757-3 (PMC13749715; doi:10.1186/s12870-026-09757-3)
Supplement: Supplementary file 1 [file 12870_2026_9757_MOESM1_ESM.docx]

**Figure S1** Overview of carotenoid biosynthesis and regulatory pathways in plants and algae

**Figure S2** Sequence logos of the conserved basic region and leucine zipper domain of LjbZIP proteins. The bit values represent the information content of each amino acid at a specific position in a sequence

**Figure S3** Distribution of predicted cis-acting regulatory elements in the promoter regions of *LjbZIP* genes

**Figure S4** Synteny analysis of the *LjbZIP* gene family. A: Syntenic relationships between *Lonicera japonica* and *Arabidopsis thaliana*. B: Syntenic relationships between *L. japonica* and *Oryza sativa*. C: Intraspecies synteny analysis of the *L. japonica* genome showing duplicated *LjbZIP* gene pairs. Colored segments represent chromosomes, gray lines indicate genome-wide syntenic blocks, and colored lines highlight syntenic relationships among *LjbZIP* genes. D: Classification of duplicated *LjbZIP* genes based on homologous cluster size. Light blue, green, and yellow colors indicate homologous groups containing two, three, and four *LjbZIP* members, respectively

**Figure S5** Correlation analysis between gene expression profiles and carotenoid metabolites based on integrated transcriptomic and metabolomic datasets across five floral developmental stages (S1–S5). S1, green buds. S2, slightly white buds. S3, whole white buds. S4, silver flowers (initial blooming, white flowers). S5, golden flowers (fully opened, yellow flowers). Rows represent genes and columns represent metabolites. Red and blue indicate positive and negative correlations, respectively. A: S1 vs S2. B: S1 vs S3. C: S1 vs S4. D: S1 vs S5. E: S2 vs S3. F: S2 vs S4. G: S2 vs S5. H: S3 vs S4. I: S3 vs S5. J: S4 vs S5

**Figure S6** Relationship between *LjbZIP36* expression and β-carotene accumulation during floral development of *Lonicera japonica* flowers from S1 to S5

**Figure S7** Representative LC–MS/MS chromatograms used for carotenoid identification and quantification. Chromatograms are shown for blank, authentic standards, empty vector (EV), and *LjbZIP36*-overexpressing (*LjbZIP36-OE*) samples. A: β-carotene. B: (E/Z)-phytoene
