# Supplementary material for: Integrative genomic and functional analyses identify LjbZIP36 as a candidate regulator of β-carotene accumulation during floral color transition in Lonicera japonica
Source: BMC Plant Biol. 2026 Aug 14;26:1926. doi: 10.1186/s12870-026-09757-3 (PMC13749715; doi:10.1186/s12870-026-09757-3)
Supplement: Supplementary file 2 [file 12870_2026_9757_MOESM2_ESM.docx]

**Table S1** Characteristics of identified *LjbZIP* genes in *Lonicera japonica*.

| Gene name | Gene ID | Transcript ID | Protein ID | Pos | Amino acid no. | Molecular weight (Da) | Isoelectric points | Instability Index |
| --- | --- | --- | --- | --- | --- | --- | --- | --- |
| *LjbZIP1* | IMPGLJA1N46621 | IMPTLJA1N46621_1 | IMPTLJA1N46621_1 | chr1:1808903-1809331 | 142 | 16337.51 | 7.87 | 47.59 |
| *LjbZIP2* | IMPGLJA1N46714 | IMPTLJA1N46714_1 | IMPTLJA1N46714_1 | chr1:24127347-24130034 | 336 | 37886.24 | 5.84 | 53.12 |
| *LjbZIP3* | IMPGLJA1N51516 | IMPTLJA1N51516_1 | IMPTLJA1N51516_1 | chr1:24171930-24182011 | 521 | 58507.23 | 6.4 | 61.28 |
| *LjbZIP4* | IMPGLJA1N50137 | IMPTLJA1N50137_1 | IMPTLJA1N50137_1 | chr1:26870841-26878738 | 329 | 36827.63 | 8.98 | 67.79 |
| *LjbZIP5* | IMPGLJA1N52706 | IMPTLJA1N52706_1 | IMPTLJA1N52706_1 | chr1:72744127-72746885 | 281 | 31738.11 | 5.77 | 71.99 |
| *LjbZIP6* | IMPGLJA1N43515 | IMPTLJA1N43515_1 | IMPTLJA1N43515_1 | chr1:85884184-85887148 | 349 | 38150.55 | 5.94 | 55.96 |
| *LjbZIP7* | IMPGLJA1N48665 | IMPTLJA1N48665_1 | IMPTLJA1N48665_1 | chr1:97515049-97518987 | 777 | 83754.51 | 5.59 | 50.4 |
| *LjbZIP8* | IMPGLJA1N42848 | IMPTLJA1N42848_1 | IMPTLJA1N42848_1 | chr1:98273058-98288093 | 471 | 51285.23 | 7.16 | 49.27 |
| *LjbZIP9* | IMPGLJA1N51492 | IMPTLJA1N51492_1 | IMPTLJA1N51492_1 | chr1:103736348-103743952 | 523 | 58557.66 | 6.8 | 60.54 |
| *LjbZIP10* | IMPGLJA1N33816 | IMPTLJA1N33816_1 | IMPTLJA1N33816_1 | chr2:13414436-13418224 | 454 | 50496.6 | 6.78 | 69.5 |
| *LjbZIP11* | IMPGLJA1N22452 | IMPTLJA1N22452_1 | IMPTLJA1N22452_1 | chr2:13691468-13693852 | 383 | 42116.4 | 7.66 | 59.74 |
| *LjbZIP12* | IMPGLJA1N33606 | IMPTLJA1N33606_1 | IMPTLJA1N33606_1 | chr2:23580287-23589384 | 398 | 42291.43 | 5.9 | 62.1 |
| *LjbZIP13* | IMPGLJA1N32823 | IMPTLJA1N32823_1 | IMPTLJA1N32823_1 | chr2:40647124-40655253 | 461 | 50824.81 | 6.22 | 55.07 |
| *LjbZIP14* | IMPGLJA1N31669 | IMPTLJA1N31669_1 | IMPTLJA1N31669_1 | chr2:41346486-41353860 | 715 | 78461.69 | 5.89 | 47.24 |
| *LjbZIP15* | IMPGLJA1N22328 | IMPTLJA1N22328_1 | IMPTLJA1N22328_1 | chr2:41994281-41995324 | 317 | 35269.72 | 7.04 | 54.29 |
| *LjbZIP16* | IMPGLJA1N28445 | IMPTLJA1N28445_1 | IMPTLJA1N28445_1 | chr2:45513126-45519679 | 367 | 39060.71 | 5.48 | 59.22 |
| *LjbZIP17* | IMPGLJA1N29867 | IMPTLJA1N29867_1 | IMPTLJA1N29867_1 | chr2:46524567-46525022 | 151 | 17128.37 | 5.35 | 49.92 |
| *LjbZIP18* | IMPGLJA1N22943 | IMPTLJA1N22943_1 | IMPTLJA1N22943_1 | chr2:48751403-48755144 | 199 | 22575.27 | 8.79 | 69.05 |
| *LjbZIP19* | IMPGLJA1N24514 | IMPTLJA1N24514_1 | IMPTLJA1N24514_1 | chr2:56296594-56303438 | 364 | 40597.9 | 7.64 | 43.25 |
| *LjbZIP20* | IMPGLJA1N30685 | IMPTLJA1N30685_1 | IMPTLJA1N30685_1 | chr2:86795646-86800964 | 258 | 29271.34 | 8.85 | 46.26 |
| *LjbZIP21* | IMPGLJA1N22721 | IMPTLJA1N22721_1 | IMPTLJA1N22721_1 | chr2:88842003-88851203 | 504 | 56175.07 | 6.54 | 61.14 |
| *LjbZIP22* | IMPGLJA1N24715 | IMPTLJA1N24715_1 | IMPTLJA1N24715_1 | chr2:101520397-101526756 | 164 | 19280.8 | 5.22 | 84.26 |
| *LjbZIP23* | IMPGLJA1N22692 | IMPTLJA1N22692_1 | IMPTLJA1N22692_1 | chr2:103034530-103036738 | 363 | 40078.97 | 5.97 | 59.61 |
| *LjbZIP24* | IMPGLJA1N78456 | IMPTLJA1N78456_1 | IMPTLJA1N78456_1 | chr3:8535163-8542108 | 436 | 48135.26 | 6.24 | 57.42 |
| *LjbZIP25* | IMPGLJA1N75651 | IMPTLJA1N75651_1 | IMPTLJA1N75651_1 | chr3:75728630-75730754 | 408 | 44866.91 | 7.16 | 52.72 |
| *LjbZIP26* | IMPGLJA1N78528 | IMPTLJA1N78528_1 | IMPTLJA1N78528_1 | chr3:78366492-78367043 | 183 | 21334.77 | 6.38 | 75.85 |
| *LjbZIP27* | IMPGLJA1N100344 | IMPTLJA1N100344_1 | IMPTLJA1N100344_1 | chr4:542192-543557 | 219 | 24890.54 | 4.5 | 56.87 |
| *LjbZIP28* | IMPGLJA1N95795 | IMPTLJA1N95795_1 | IMPTLJA1N95795_1 | chr4:2199644-2200887 | 281 | 31549.62 | 10.24 | 88.75 |
| *LjbZIP29* | IMPGLJA1N95979 | IMPTLJA1N95979_1 | IMPTLJA1N95979_1 | chr4:3721128-3721622 | 164 | 19055.27 | 5.61 | 71.31 |
| *LjbZIP30* | IMPGLJA1N101839 | IMPTLJA1N101839_1 | IMPTLJA1N101839_1 | chr4:6217414-6221643 | 476 | 51321.68 | 9.41 | 46.73 |
| *LjbZIP31* | IMPGLJA1N93146 | IMPTLJA1N93146_1 | IMPTLJA1N93146_1 | chr4:7805060-7805476 | 138 | 16395.36 | 8.93 | 64.53 |
| *LjbZIP32* | IMPGLJA1N102333 | IMPTLJA1N102333_1 | IMPTLJA1N102333_1 | chr4:13262348-13262938 | 196 | 23159.52 | 5.98 | 64.85 |
| *LjbZIP33* | IMPGLJA1N101011 | IMPTLJA1N101011_1 | IMPTLJA1N101011_1 | chr4:17083213-17083887 | 195 | 22505.42 | 9.93 | 61.63 |
| *LjbZIP34* | IMPGLJA1N97756 | IMPTLJA1N97756_1 | IMPTLJA1N97756_1 | chr4:20068670-20073561 | 355 | 37871.52 | 6.21 | 59.58 |
| *LjbZIP35* | IMPGLJA1N99277 | IMPTLJA1N99277_1 | IMPTLJA1N99277_1 | chr4:58736360-58739638 | 359 | 41117.74 | 6.47 | 47.42 |
| *LjbZIP36* | IMPGLJA1N98341 | IMPTLJA1N98341_1 | IMPTLJA1N98341_1 | chr4:64706383-64711317 | 438 | 47416.02 | 9.76 | 50.36 |
| *LjbZIP37* | IMPGLJA1N97743 | IMPTLJA1N97743_1 | IMPTLJA1N97743_1 | chr4:66500686-66501150 | 154 | 18020.5 | 6.31 | 57.89 |
| *LjbZIP38* | IMPGLJA1N96499 | IMPTLJA1N96499_1 | IMPTLJA1N96499_1 | chr4:75430448-75440581 | 361 | 41003.63 | 6.28 | 48.69 |
| *LjbZIP39* | IMPGLJA1N16714 | IMPTLJA1N16714_1 | IMPTLJA1N16714_1 | chr5:16875788-16877021 | 292 | 31867.55 | 6.31 | 32.33 |
| *LjbZIP40* | IMPGLJA1N16059 | IMPTLJA1N16059_1 | IMPTLJA1N16059_1 | chr5:18441970-18442428 | 152 | 16775.84 | 6.42 | 51.66 |
| *LjbZIP41* | IMPGLJA1N14123 | IMPTLJA1N14123_1 | IMPTLJA1N14123_1 | chr5:20050333-20053195 | 440 | 47909.83 | 9.55 | 48.99 |
| *LjbZIP42* | IMPGLJA1N12321 | IMPTLJA1N12321_1 | IMPTLJA1N12321_1 | chr5:23757307-23759621 | 265 | 29186.82 | 7.03 | 66.99 |
| *LjbZIP43* | IMPGLJA1N10283 | IMPTLJA1N10283_1 | IMPTLJA1N10283_1 | chr5:31770351-31770785 | 144 | 16678.21 | 9.26 | 71.46 |
| *LjbZIP44* | IMPGLJA1N109809 | IMPTLJA1N109809_1 | IMPTLJA1N109809_1 | chr6:7084404-7090429 | 435 | 47993.41 | 6.31 | 65.5 |
| *LjbZIP45* | IMPGLJA1N109219 | IMPTLJA1N109219_1 | IMPTLJA1N109219_1 | chr6:49372432-49372875 | 147 | 17201.33 | 7.86 | 66.25 |
| *LjbZIP46* | IMPGLJA1N104922 | IMPTLJA1N104922_1 | IMPTLJA1N104922_1 | chr6:56584075-56588745 | 483 | 52798.94 | 6.1 | 45.58 |
| *LjbZIP47* | IMPGLJA1N108663 | IMPTLJA1N108663_1 | IMPTLJA1N108663_1 | chr6:57459053-57459631 | 192 | 22182.7 | 5.97 | 71.99 |
| *LjbZIP48* | IMPGLJA1N90846 | IMPTLJA1N90846_1 | IMPTLJA1N90846_1 | chr7:8168419-8168877 | 152 | 18261.3 | 5.85 | 65.39 |
| *LjbZIP49* | IMPGLJA1N84319 | IMPTLJA1N84319_1 | IMPTLJA1N84319_1 | chr7:17331570-17332145 | 191 | 22400.06 | 10.21 | 72.09 |
| *LjbZIP50* | IMPGLJA1N88554 | IMPTLJA1N88554_1 | IMPTLJA1N88554_1 | chr7:37601892-37608042 | 353 | 39038.96 | 7.12 | 44.82 |
| *LjbZIP51* | IMPGLJA1N86826 | IMPTLJA1N86826_1 | IMPTLJA1N86826_1 | chr7:37938354-37943227 | 285 | 31560.42 | 6.14 | 64.41 |
| *LjbZIP52* | IMPGLJA1N83378 | IMPTLJA1N83378_1 | IMPTLJA1N83378_1 | chr7:50818929-50823042 | 407 | 43391.53 | 6.7 | 48.47 |
| *LjbZIP53* | IMPGLJA1N83022 | IMPTLJA1N83022_1 | IMPTLJA1N83022_1 | chr7:76576230-76581423 | 335 | 36158.92 | 5.38 | 48.44 |
| *LjbZIP54* | IMPGLJA1N2415 | IMPTLJA1N2415_1 | IMPTLJA1N2415_1 | chr8:4278359-4280338 | 301 | 33731.44 | 5.81 | 68.95 |
| *LjbZIP55* | IMPGLJA1N6845 | IMPTLJA1N6845_1 | IMPTLJA1N6845_1 | chr8:9195411-9197913 | 291 | 32749.51 | 5.73 | 60.64 |
| *LjbZIP56* | IMPGLJA1N6827 | IMPTLJA1N6827_1 | IMPTLJA1N6827_1 | chr8:14574643-14579613 | 624 | 70773.21 | 8.86 | 34.52 |
| *LjbZIP57* | IMPGLJA1N817 | IMPTLJA1N817_1 | IMPTLJA1N817_1 | chr8:20824220-20830630 | 348 | 38612.8 | 6.1 | 50.62 |
| *LjbZIP58* | IMPGLJA1N6965 | IMPTLJA1N6965_1 | IMPTLJA1N6965_1 | chr8:30748514-30756899 | 389 | 41193.13 | 5.58 | 60.56 |
| *LjbZIP59* | IMPGLJA1N5237 | IMPTLJA1N5237_1 | IMPTLJA1N5237_1 | chr8:57721126-57724108 | 160 | 17779.82 | 9.64 | 67.55 |
| *LjbZIP60* | IMPGLJA1N64228 | IMPTLJA1N64228_1 | IMPTLJA1N64228_1 | chr9:41787575-41793315 | 267 | 29105.74 | 5.48 | 56.71 |
| *LjbZIP61* | IMPGLJA1N59645 | IMPTLJA1N59645_1 | IMPTLJA1N59645_1 | chr9:48211851-48216951 | 580 | 63271.56 | 6.36 | 62.52 |
| *LjbZIP62* | IMPGLJA1N59539 | IMPTLJA1N59539_1 | IMPTLJA1N59539_1 | chr9:49120265-49121035 | 256 | 28362.7 | 5.73 | 31.73 |
| *LjbZIP63* | IMPGLJA1N67450 | IMPTLJA1N67450_1 | IMPTLJA1N67450_1 | chr9:73542216-73542866 | 216 | 23997.77 | 5.81 | 40.53 |
| *LjbZIP64* | IMPGLJA1N58660 | IMPTLJA1N58660_1 | IMPTLJA1N58660_1 | chr9:75564633-75575075 | 435 | 47215.26 | 5.75 | 62.63 |
| *LjbZIP65* | IMPGLJA1N92644 | IMPTLJA1N92644_1 | IMPTLJA1N92644_1 | JAKCWG010000360:76606-77504 | 128 | 14691.98 | 9.97 | 43.41 |
| *LjbZIP66* | IMPGLJA1N81376 | IMPTLJA1N81376_1 | IMPTLJA1N81376_1 | JAKCWG010000561:7855-12756 | 355 | 37772.38 | 6.03 | 59.1 |
| *LjbZIP67* | IMPGLJA1N57 | IMPTLJA1N57_1 | IMPTLJA1N57_1 | JAKCWG010000762:61748-65195 | 117 | 13972.26 | 10.01 | 44.97 |
| *LjbZIP68* | IMPGLJA1N17 | IMPTLJA1N17_1 | IMPTLJA1N17_1 | JAKCWG010000762:440813-443794 | 160 | 17779.82 | 9.64 | 67.55 |

**Table S2** bZIP transcription factors from *Arabidopsis thaliana* used for phylogenetic analysis.

| Gene name | Gene ID |
| --- | --- |
| *AtABF1* | AT1G49720 |
| *AtABF2* | AT1G45249 |
| *AtbZIP1* | AT5G49450 |
| *AtbZIP10* | AT4G02640 |
| *AtbZIP11* | AT4G34590 |
| *AtbZIP12* | AT2G41070 |
| *AtbZIP13* | AT5G44080 |
| *AtbZIP14* | AT4G35900 |
| *AtbZIP15* | AT5G42910 |
| *AtbZIP16* | AT2G35530 |
| *AtbZIP17* | AT2G40950 |
| *AtbZIP18* | AT2G40620 |
| *AtbZIP19* | AT4G35040 |
| *AtbZIP2* | AT2G18160 |
| *AtbZIP20* | AT5G06950 |
| *AtbZIP21* | AT1G08320 |
| *AtbZIP22* | AT1G22070 |
| *AtbZIP23* | AT2G16770 |
| *AtbZIP24* | AT3G51960 |
| *AtbZIP25* | AT3G54620 |
| *AtbZIP26* | AT5G06960 |
| *AtbZIP27* | AT2G17770 |
| *AtbZIP28* | AT3G10800 |
| *AtbZIP29* | AT4G38900 |
| *AtbZIP3* | AT5G15830 |
| *AtbZIP30* | AT2G21230 |
| *AtbZIP31* | AT2G13150 |
| *AtbZIP33* | AT2G12900 |
| *AtbZIP34* | AT2G42380 |
| *AtbZIP37* | AT4G34000 |
| *AtbZIP38* | AT3G19290 |
| *AtbZIP39* | AT2G36270 |
| *AtbZIP4* | AT1G59530 |
| *AtbZIP40* | AT1G03970 |
| *AtbZIP41* | AT4G36730 |
| *AtbZIP42* | AT3G30530 |
| *AtbZIP43* | AT5G38800 |
| *AtbZIP44* | AT1G75390 |
| *AtbZIP45* | AT3G12250 |
| *AtbZIP46* | AT1G68640 |
| *AtbZIP47* | AT5G65210 |
| *AtbZIP48* | AT2G04038 |
| *AtbZIP49* | AT3G56660 |
| *AtbZIP5* | AT3G49760 |
| *AtbZIP50* | AT1G77920 |
| *AtbZIP51* | AT1G43700 |
| *AtbZIP52* | AT1G06850 |
| *AtbZIP53* | AT3G62420 |
| *AtbZIP54* | AT4G01120 |
| *AtbZIP55* | AT2G46270 |
| *AtbZIP56* | AT5G11260 |
| *AtbZIP57* | AT5G10030 |
| *AtbZIP58* | AT1G13600 |
| *AtbZIP59* | AT2G31370 |
| *AtbZIP6* | AT2G22850 |
| *AtbZIP60* | AT1G42990 |
| *AtbZIP61* | AT3G58120 |
| *AtbZIP62* | AT1G19490 |
| *AtbZIP63* | AT5G28770 |
| *AtbZIP66* | AT3G56850 |
| *AtbZIP67* | AT3G44460 |
| *AtbZIP68* | AT1G32150 |
| *AtbZIP69* | AT1G06070 |
| *AtbZIP7* | AT4G37730 |
| *AtbZIP70* | AT5G60830 |
| *AtbZIP71* | AT2G24340 |
| *AtbZIP72* | AT5G07160 |
| *AtbZIP74* | AT2G21235 |
| *AtbZIP8* | AT1G68880 |
| *AtbZIP9* | AT5G24800 |

**Table S3** Primer sequences used in this study.

| Primers | Primer sequence (5’to3’) | Application |
| --- | --- | --- |
| LjA1N98341-5 | 5'- GAT ATG TTG AAT CCT GGG -3' | Quantitative real-time PCR |
| LjA1N98341-6 | 5'- CGT AGT TCT AAT CCC ATC AAC -3' |  |
| LjG6PD-F | 5’- GAC CCA ACA GTT CCT GACAA -3' |  |
| LjG6PD-R | 5’- GCTTTCCCT GCC TTG AGTATA A -3' |  |
| Nb Actin-F | 5'- AAGGTAGTCGCACCACCAGAGAG -3' |  |
| Nb Actin-R | 5'-TGACTCGTCGTACTCTGCCTTTG-3' |  |
| Nbe-LCYB1-3 | 5'- CCA GAG TCT TTA GAT GTT G -3' |  |
| Nbe-LCYB1-4 | 5'- CAT AGG CCA CTT ATG ATC -3' 54.1 ºC |  |
| Nbe-PSY1-3 | 5'- CTT GTG AGG TCT CAA ATG -3' |  |
| Nbe-PSY1-4 | 5'- CAT CTG TTC TGA TGT CAT C -3' |  |
| Nbe-PDS-3 | 5'- GAG TCC AAG GTA ATT CAG -3' |  |
| Nbe-PDS-4 | 5'- GTC AAT CTT CTG GTC ATG -3' |  |
| Nbe-ZDS-7 | 5'- CTA CTT CAG CTA CTG GAA AG -3' |  |
| Nbe-ZDS-8 | 5'- CAC GAA TAA CTG ACT TCC -3' |  |
| LjbZIP67-1 | 5'- gattatgcctctcccgaattcATGAACTTCAAGAACTTTGGGAATG -3' | Yeast one-hybrid assay |
| LjbZIP67-2 | 5'- agaagtccaaagcttctcgagTCACCACATCCCCGTCTGC -3' |  |
| LjZDS-5 | 5'- cttgaattcgagctcggtaccCGATCCACCTTGGCTTTTGT -3' |  |
| LjZDS-6 | 5'- atacagagcacatgcctcgagGAATTTAACTAACTATGCAAGTAAACACAAT -3' |  |
| Lj-PSY2-6 | 5'- cttgaattcgagctcggtaccGATTTTCGAGTTCAATTCTAACATTATAAT -3' |  |
| Lj-PSY2-7 | 5'- atacagagcacatgcctcgagCTTGGACTGGAAAATTTTGGTTC -3' |  |
| LjPSY1-1 | 5'- cttgaattcgagctcggtaccATTATGGTATGATTATTTAGTTGCATCTTATT -3' |  |
| LjPSY1-2 | 5'- atacagagcacatgcctcgagGATGGGCTTAAAATTTGGTAACTCT -3' |  |
| LjPDS-5 | 5'- cttgaattcgagctcggtaccTGGAGATTTGTTCCATTGATCTAGA -3' |  |
| LjPDS-6 | 5'- atacagagcacatgcctcgagGGTGATGAGGTTTTGTGGGTGT -3' |  |
| LjLCYB1-5 | 5'- cttgaattcgagctcggtaccCAACCATATAATTTATTAATGTTAAATTGTGA -3' |  |
| LjLCYB1-6 | 5'- atacagagcacatgcctcgagAACAATAAAATGGAGTGTTTGTTAGAGA -3' |  |
| LjPDS-1 | 5'- gtcgacggtatcgataagcttTGGAGATTTGTTCCATTGATCTAGA -3' | Dual-luciferase reporter assay |
| LjPDS-2 | 5'- cgctctagaactagtggatccGGTGATGAGGTTTTGTGGGTGT -3' |  |
| LjZDS-1 | 5'- gtcgacggtatcgataagcttCGATCCACCTTGGCTTTTGT -3' |  |
| LjZDS-2 | 5'- cgctctagaactagtggatccGAATTTAACTAACTATGCAAGTAAACACAAT -3' |  |
| LjLCYB1-1 | 5'- gtcgacggtatcgataagcttCAACCATATAATTTATTAATGTTAAATTGTGA -3' |  |
| LjLCYB1-2 | 5'- cgctctagaactagtggatccAACAATAAAATGGAGTGTTTGTTAGAGA -3' |  |
| LjPSY1-F-pGreen0800 | 5'- ctatagggcgaattgggtaccATTATGGTATGATTATTTAGTTGCATCTTATTTA-3' |  |
| LjPSY1-R-pGreen0800 | 5'- cgctctagaactagtggatccGATGGGCTTAAAATTTGGTAACTCTA-3' |  |
| Lj-PSY2-6 | 5'- cttgaattcgagctcggtaccGATTTTCGAGTTCAATTCTAACATTATAAT -3' |  |
| Lj-PSY2-7 | 5'- atacagagcacatgcctcgagCTTGGACTGGAAAATTTTGGTTC -3' |  |
| LjA1N98341-EcoRI-F | 5'- cgggggactcttgacgaattcATGAACTTCAAGAACTTTGGGAATG -3' | Overexpression in Nicotiana benthamiana |
| LjA1N98341-BamHI-R | 5'- gagctggtcaccctaggatccTCACCACATCCCCGTCTGC -3' |  |
| LjA1N98341-HindIII-F | 5'- caaatcgactctagaaagcttATGAACTTCAAGAACTTTGGGAATG -3' | Subcellular Localization in Nicotiana benthamiana |
| LjA1N98341-KpnI-R | 5'- gcccttgctcaccatggtaccCCACATCCCCGTCTGCGT -3' |  |

**Table S4** bZIP proteins used for phylogenetic and comparative analyses of LjbZIP36.

| Metabolite | Gene name | Gene ID | Species | Protein sequence |
| --- | --- | --- | --- | --- |
| Flavonoid | VvbZIPC22 | Vitvi07g00413 | *Vitis vinifera* | MSAMQQQLGSAPNWGEPKIAVIDDRKRKRMQSNRESAKRSRIRKQQHLDDLLSKAAQLQKENGQIAERIDKTTELYIKIASDNNVLNAQIVELTDRLQSLNSVLQIVEEVNGLAVDIPEMPDDLLEPWQLPCPMPIMYPANMFQC |
|  | GmbZIP131 | NM_001250265 | *Glycine max* | MNQKFIGKPPATVDLDRMPERGSHHRRSHSDTSFRFAANFDDLLLFDPSDFDISGLPSPLPLPSPVSAGLVPMSVDSDESGKQPRPAGASAGGHLRSLSVDSDFFDGLGFGGDERGAGKGGGGERRVGHHRHSNSMDGSSTTSFEADSAMIMDGVKKAMAPDKLAELALMDPKRAKRILANRQSAARSKERKIRYTSELERKVQTLQTEATNLSAQLTMLQRDTTDLTTENKELKLRLEALEQEAQLREDLNEALKEELQRLRAQSTRLGAIGGNPSFGGIFNQLASQLAMQQLSNSAPHQQTQHQPQVGMPPPPSGQNHPNFMDFNQQK |
| Lignin | PtrBPL1 | Potri.016G040500 | *Populus trichocarpa* | MTLEDSLRTLSLDYLNLLINGQAFSDVTFSVEGRLVHAHRCILAARSLFFRKYFCGPDPPSGLDPSGSRINLVGSPGSRSNVIPVNSVGYEVFLLLLQFLYSGQVSIVPQKHEPRPNCGVRGCWHTHCTSAVDLALDTLAAARYFGVEQLAMLTQKQLASMVDNASIDDVMKVLIASRKQDMHQLWTTCSHLVAKSGLPPEVLAKHLPIDVVAKIEELRLKSSIARRSLMPHHHHHLHDLTAAADLEDQKIRRMKRALDSSDVELVKLMVMGEGLNLNEALALHYAVENCSREVVKALLELGAADVNYPAGPAGKTPLHIAAEMVSPDMVSVLLDHHADPNVRTVDGITPLDILRTLTSDFLFKGAVPGLVHIEPNKLRLCLELVQSAASVLSREEGSVNATTSNPIYPPMSDEHNSSHSSQRDHHAMSRHDPTMYRHHSHDF* |
|  | PtrBPL2 | Potri.006G043400 | *Populus trichocarpa* | MTLEDSLRSLSLDYLNLLINGQAFSDVTFSVEGRLVHAHRCILAARSLFFRKFFCGPDPPSGLDPSGSRINTVGSPGSRSNVIPVNSVGYEVFLLLLQFLYSGQVSIVPQKHEPRPNCGERGCWHTHCTSAVDLALDTLAAARYFGVEQLAMLTQKQLANMVEKASIEDVMKVLIASRKQDMHQLWTTCSHLVAKSGLPPEVLAKHLPIDVVAKIEELRLKSSIARRSLMPHHHHHLHDLTSAADLEDQKIRRMKRALDSSDVELVKLMVMGEGLNLDEALALHYAVENCSREVVKALLELGAANVNYQAGPAGKTPLHIAAEMVSPDMVAVLLDHHADPNVRTVDGVTPLDILRTLTSDFLFKGAVPGLAHMEPNKLRLCLELVQSAAMVLSREEGNVNATSTLIYPSMSDEHNTSSSGSNLANLNLDSRLVYLNLGAAGSGQMGSRMDEEDDSNHNNQRDHHAMSRHDPTMYHHHSHDF* |
|  | AtTGA1 | AT5G65210 | *Arabidopsis thaliana* | MNSTSTHFVPPRRVGIYEPVHQFGMWGESFKSNISNGTMNTPNHIIIPNNQKLDNNVSEDTSHGTAGTPHMFDQEASTSRHPDKIQRRLAQNREAARKSRLRKKAYVQQLETSRLKLIQLEQELDRARQQGFYVGNGIDTNSLGFSETMNPGIAAFEMEYGHWVEEQNRQICELRTVLHGHINDIELRSLVENAMKHYFELFRMKSSAAKADVFFVMSGMWRTSAERFFLWIGGFRPSDLLKVLLPHFDVLTDQQLLDVCNLKQSCQQAEDALTQGMEKLQHTLADCVAAGQLGEGSYIPQVNSAMDRLEALVSFVNQADHLRHETLQQMYRILTTRQAARGLLALGEYFQRLRALSSSWATRHREPT |
|  | AtTGA3 | AT1G22070 | *Arabidopsis thaliana* | MEMMSSSSSTTQVVSFRDMGMYEPFQQLSGWESPFKSDINNITSNQNNNQSSSTTLEVDARPEADDNNRVNYTSVYNNSLEAEPSSNNDQDEDRINDKMKRRLAQNREAARKSRLRKKAHVQQLEESRLKLSQLEQELVRARQQGLCVRNSSDTSYLGPAGNMNSGIAAFEMEYTHWLEEQNRRVSEIRTALQAHIGDIELKMLVDSCLNHYANLFRMKADAAKADVFFLMSGMWRTSTERFFQWIGGFRPSELLNVVMPYVEPLTDQQLLEVRNLQQSSQQAEEALSQGLDKLQQGLVESIAIQIKVVESVNHGAPMASAMENLQALESFVNQADHLRQQTLQQMSKILTTRQAARGLLALGEYFHRLRALSSLWAARPREHT |
|  | AtTGA4 | AT5G10030 | *Arabidopsis thaliana* | MNTTSTHFVPPRRFEVYEPLNQIGMWEESFKNNGDMYTPGSIIIPTNEKPDSLSEDTSHGTEGTPHKFDQEASTSRHPDKIQRRLAQNREAARKSRLRKKAYVQQLETSRLKLIHLEQELDRARQQGFYVGNGVDTNALSFSDNMSSGIVAFEMEYGHWVEEQNRQICELRTVLHGQVSDIELRSLVENAMKHYFQLFRMKSAAAKIDVFYVMSGMWKTSAERFFLWIGGFRPSELLKVLLPHFDPLTDQQLLDVCNLRQSCQQAEDALSQGMEKLQHTLAESVAAGKLGEGSYIPQMTCAMERLEALVSFVNQADHLRHETLQQMHRILTTRQAARGLLALGEYFQRLRALSSSWAARQREPT |
|  | AtTGA7 | AT1G77920 | *Arabidopsis thaliana* | MMSSSSPTQLASLRDMGIYEPFQQIVGWGNVFKSDINDHSPNTATSSIIQVDPRIDDHNNNIKINYDSSHNQIEAEQPSSNDNQDDDGRIHDKMKRRLAQNREAARKSRLRKKAYVQQLEESRLKLSQLEQELEKVKQQGHLGPSGSINTGIASFEMEYSHWLQEQSRRVSELRTALQSHISDIELKMLVESCLNHYANLFQMKSDAAKADVFYLISGMWRTSTERFFQWIGGFRPSELLNVVMPYLQPLTDQQILEVRNLQQSSQQAEDALSQGIDKLQQSLAESIVIDAVIESTHYPTHMAAAIENLQALEGFVNQADHLRQQTLQQMAKILTTRQSARGLLALGEYLHRLRALSSLWAARPQEPT |
| Anthocyanin | MdABI5 | MD14G1021600 | *Malus domestica* | MGVLESEINSHGEVDSPLLSDQQAKNHLFSSLGRQSSIYSLTLDEFQHTLCESGKNFGLMNMDEFLTSIWTAEENQAINSNLTNTTTTNNNNNMNNIEVHIPLAEASAEKSIATQPSLPRQGSLTLPEPLCRKTVDEVWSEINGGQQAKQQNNHNSSIDGSVQSSEFAPRQPTFGEMTLEDFLVKAGVVREQDSMAATVVPPQPHQQQRYGMYQNGNHAVGPSFVNRPVMGMGAAAAAGASTSTAAGIPNYQTIPQSGAAVVGESSAYAANGKRNGAYPGVRPPQSVCFGGRVENGGGGYAAGQTIGMAAPGSPVSSDGMGTSQVENSAGRFGLDMGLDGLRGRKRGLDGPVEKVVERRQRRMIKNRESAARSRARKQAYTVELEAELNHLREENSHLKQALSSRGSESNSISRK |
|  | HlbZIP1 | CAZ15514.1 | *Humulus lupulus L* | MALSNVVIASASATNSELPRQSSICSPNTIMSEEDSRNIASMDDLLKNIPNIFPEQPQQQQQQNSHPLASFPVAGVGESWSGAVASGSDWKTDSGEIGSAMTLEDYLTHSEAVREEDIRAQLGYGQFHMPLQLQAVENPVVVCGNGSGTSGRGKRQAVDPPVDKATAQKQRRMIKNRESAARSRERKQAYQVELETLVTELEDEKARLLREEVERTQERFRQLMKNVIPVEEKKKTPRVLRRVRSL |
|  | HlbZIP2 | CAQ53097.1 | *Humulus lupulus L* | MASSKVLASSNSRNSDLSRKSSSASSSAVRRPKLSFSDQQAHIINSNNNGGDHTAKPSMTVDGFLRNVYDATPAAESTLLDAQITLIDPTPIASVSAAAVATGDLNSGSIGSSSAPKTVDEVWREIISGDRKECKEEEQDMVMTLEDFLLAKTGIASVEEEDVKSLPAPLTESLSSGLFSFDSIPPSPLQALDNVEGSIIGFGNGVEVIGGGGAGGRGKRGRNVLEPLDKAAQQRQRRMIKNRESAARSRERKQAYQVELESLAVRLEEENDRLLKEKAERTKERFKQLMEKVIPVEEKRRPPRVLRRVRSLQW |
|  | HvABI5 | HQ456390.1 | *Hordeum vulgare* | MDFRSSNGGSSSERRPAAEGASLTRQGSIYSLTFEEFQSTLGGSAGVGGGDLGKDFSSMNMDELLRSIWTAEESQAMAASASGAGAGAPPMSLQGQGSLTLPRTLSAKTVDEVWRNLVRDDPLPVGAEGAEPQPHRQATLGEMTLEEFLVKAGVVREIPTAPAVPPPPMQPRPVPVAPKGATFYGNFPSANDVGTAALGFPPVAMGDLALGNGLMPRALGMGGAPLVVQTAVKPVDSGSKGSEDLSSPSEPMPYSFEGIVRGRRTGGGVEKVVERRQRRMIKNRESAARSRARKQAYTMELEAEVQKLKDLNEELVKKQTEILKMQKREAPEMKDQFGRKKRQCLRRTLTGW |
| Carotenoid | SlAREB1 | AY530758 | *Solanum lycopersicum* | MGSNYHFKNFGNEPPGEGGSGGGGKQPGNFGLPRQPSIYSLTFDEFLSSTGGSGKDFGSMNMDELLKNIWNAEENQTIGGPGINGQEVGVPGGHLQRQGSLTLPRTLSHKTVDEVWRDMSKEHGGGKDGNSVGVPPNIPQTQRQQNLGEITLEEFLVRAGVVREDAQFAAKSNNAGGIFGDLSYAGNNTGLAFGYHQANSRNTGLMAGSIPNKNGETVIQSANLPLNVNGVRSTQQQLRPQQLQQNQQSQPQQQPIFPKQPALPYGAPMAIPNSGQLGSPGMRAGMVGIPDPALNSNFIQGASLMGGGMNMVGLGASGVTVATASPGVSSSDGLGKSNGDTPSVSPVPYVFNGGLRGRKYSTVEKVVERRQRRMIKNRESAARSRARKQAYTMELEAEVAKLKEENDELQKKQEEMLEMQ  KNQVIEMMNLQKGAKRRCLRRTQTGPW |
|  | MibZIP66 | Mango_gene35447 | *Mangifera indica* | MGIQTMGSQGDGSNSGKQTQFQPLVRQNSMYSLTLDEVQNQLGDLGKPLTSMNLDELLKNVWTVEANQSMSVEMEGAASANQTALQHQASLSLTGALSKKTVDEVWRDIQQNKNSGERKSRERQPTLGEMTLEDFLVKAGVVAEASTDKKTGGPIGIDQNLTPQFPQGQWMQYQHPQYQQSQQSMMGVYMSGQPIPQPLHMGSGAIMDVSFSDNQVSLTSPSMGTLSDIQTLGRKRGTPEEIIEKTVERRQKRMIKNRESAARSRARKQAYTNELEIKVSRLEEENERLRKWKELEQLLPSAMPPEPKYQLRRTTSASF* |
|  | LjbZIP36 | IMPTLJA1N98341_1 | *Lonicera japonica* | MNFKNFGNEPPGDGGRSQGNFSLARQTSIYTLTFDELQNTIGGSGKDFGSMNMDELLKNIWSAEETQTMGSANTGVQQGVAPGPGGYLQKQGSLTLPRTLSQKTVDEVWRDMSNGGKDVSGFGGLNLQQRQQTLGEMTLEEFLVKAGVVREENLFTGKPDNMSGLFGNDMLNPGNNPAFGMGFQQTGQNTGLVGNRIPESNNNNNQFSFQSANLPLNVDGIRTTQQQQQQQQQQQQQQQQQQQQQLLPKRPTMTYASPMPMPITAQLGTPGTRGGIMGISDSGLNSSLAQNVGLQGGGTGRVGGGGIGAVTVAAAASPVVSSNGFGKSNGDISSVSPVPYVFNGGLRGRKGNGVEKVVERRQRRMIKNRESAARSRARKQAYTMELEAEIAKLKEENEDLQKKQAEITKMQKNQIVEMANMQKGAKRRSLRRTQTGMW |

**Table S5** The FPKM values of *LjbZIP* genes.

| Gene name | S1-1 | S1-2 | S1-3 | S2-1 | S2-2 | S2-3 | S3-1 | S3-2 | S3-3 | S4-1 | S4-2 | S4-3 | S5-1 | S5-2 | S5-3 |
| --- | --- | --- | --- | --- | --- | --- | --- | --- | --- | --- | --- | --- | --- | --- | --- |
| *LjbZIP1* | 51.33 | 55.89 | 51.5 | 50.6 | 46.13 | 51.02 | 47.76 | 49.58 | 46.99 | 39.3 | 38.22 | 39.04 | 32.37 | 35.04 | 35.54 |
| *LjbZIP2* | 0 | 0 | 0 | 0 | 0 | 0 | 0 | 0 | 0 | 0 | 0 | 0 | 0 | 0 | 0 |
| *LjbZIP3* | 7.21 | 8.42 | 7.46 | 6.05 | 4.56 | 5.85 | 3.07 | 6.55 | 2.74 | 0.4 | 0.44 | 0.45 | 0.32 | 0.24 | 0.41 |
| *LjbZIP4* | 41.6 | 36.86 | 38.2 | 33.09 | 24.18 | 35 | 30.02 | 30.45 | 30.82 | 31.87 | 32.21 | 22.76 | 23.91 | 25.03 | 23.64 |
| *LjbZIP5* | 3.65 | 3.93 | 5 | 4.69 | 5 | 4.55 | 1.88 | 3.41 | 1.72 | 0.26 | 0.32 | 0.14 | 0.07 | 0.13 | 0.38 |
| *LjbZIP6* | 16.15 | 13.81 | 13.42 | 22.41 | 17.27 | 22.36 | 19.42 | 15.57 | 18.57 | 0.97 | 1.07 | 0.95 | 0.18 | 0.47 | 0.36 |
| *LjbZIP7* | 24.41 | 25.24 | 24.51 | 41.19 | 41.88 | 42.47 | 22.46 | 22.21 | 20.26 | 11.3 | 10.32 | 10.76 | 9.66 | 8.94 | 8.3 |
| *LjbZIP8* | 35.4 | 34.7 | 34.1 | 34.09 | 27.81 | 34.55 | 34.27 | 32.23 | 30.82 | 39.25 | 37.49 | 31.82 | 27.84 | 29.54 | 29.04 |
| *LjbZIP9* | 1.1 | 1.05 | 1.01 | 0.21 | 0.24 | 0.24 | 0.18 | 0.75 | 0.31 | 0.25 | 0.1 | 0.11 | 0.12 | 0.14 | 0.38 |
| *LjbZIP10* | 3.36 | 3.24 | 2.42 | 17.18 | 27.24 | 16.55 | 5.95 | 3.41 | 4.97 | 2.66 | 2.7 | 3.5 | 3.51 | 3.07 | 3.46 |
| *LjbZIP11* | 1.06 | 0.66 | 0.57 | 1.16 | 0.97 | 0.91 | 16.28 | 9.7 | 16.76 | 45.4 | 43.76 | 57.08 | 21.52 | 18.34 | 20.5 |
| *LjbZIP12* | 33.66 | 31.58 | 33.19 | 29.33 | 28.18 | 32.72 | 31.78 | 32.23 | 28.98 | 34.45 | 36.09 | 28.53 | 31.27 | 29.76 | 30.72 |
| *LjbZIP13* | 12.03 | 11.83 | 11.95 | 14.14 | 14.28 | 15.25 | 11.73 | 11.07 | 12.23 | 5.2 | 5.9 | 4.09 | 6.83 | 7.62 | 7.17 |
| *LjbZIP14* | 27.48 | 27.45 | 26.94 | 25.81 | 37.03 | 26.36 | 25.68 | 27.62 | 24.94 | 26.25 | 25.98 | 33.39 | 34.56 | 32.4 | 33.24 |
| *LjbZIP15* | 36.68 | 34.63 | 34.72 | 34.21 | 29.49 | 35.88 | 34.94 | 33.5 | 33.82 | 24.72 | 23.06 | 17.25 | 19.72 | 19.3 | 19.19 |
| *LjbZIP16* | 27.16 | 23.59 | 24.23 | 23.77 | 22.06 | 24.94 | 25.18 | 23.67 | 26.81 | 33.11 | 30.14 | 27.25 | 29.39 | 30.39 | 32.29 |
| *LjbZIP17* | 0.21 | 0.28 | 0.41 | 0.09 | 0 | 0 | 0.11 | 0.12 | 0.27 | 1.75 | 1.64 | 2.33 | 5.19 | 3.7 | 5.89 |
| *LjbZIP18* | 0.23 | 0 | 0.16 | 0.2 | 0 | 0.14 | 0.08 | 0 | 0 | 0 | 0 | 0 | 0 | 0 | 0 |
| *LjbZIP19* | 6.16 | 5.27 | 6.16 | 5.4 | 6.1 | 5.27 | 4.52 | 5.66 | 4.92 | 5.13 | 4.88 | 4.47 | 1.14 | 1.64 | 1.27 |
| *LjbZIP20* | 36.36 | 31.08 | 37.83 | 32.68 | 36.1 | 33.5 | 35.28 | 39.34 | 39.66 | 40.15 | 40.96 | 44.18 | 46.73 | 47.52 | 44.22 |
| *LjbZIP21* | 0.12 | 0.14 | 0.15 | 0.08 | 0.11 | 0.08 | 0.16 | 0.28 | 0.28 | 1.34 | 1.48 | 1.95 | 1.56 | 1.58 | 1.42 |
| *LjbZIP22* | 0 | 0 | 0 | 0 | 0 | 0.08 | 0.1 | 0.11 | 0.12 | 0.36 | 0 | 0 | 0.13 | 0.22 | 0.11 |
| *LjbZIP23* | 46.46 | 41.41 | 46.39 | 53.44 | 29.18 | 51.66 | 73.51 | 61.86 | 74.45 | 11.71 | 12.13 | 8.8 | 8.78 | 6.38 | 7.72 |
| *LjbZIP24* | 18.98 | 19.48 | 19.59 | 21.34 | 20.83 | 20.78 | 12.44 | 15.21 | 11.5 | 9.52 | 9.04 | 8.01 | 7.93 | 6.44 | 6.35 |
| *LjbZIP25* | 0 | 0.03 | 0 | 0.03 | 0.1 | 0.1 | 0.04 | 0.04 | 0.05 | 0.04 | 0 | 0 | 0 | 0 | 0 |
| *LjbZIP26* | 1.78 | 2.06 | 2.11 | 2.93 | 2.33 | 2.8 | 1.13 | 1.55 | 0.66 | 0.32 | 0.29 | 0.21 | 0.34 | 0.2 | 0.49 |
| *LjbZIP27* | 61.12 | 57.84 | 58 | 52.4 | 44.51 | 55.29 | 45.47 | 60.97 | 50.63 | 43.38 | 44.26 | 45.13 | 44.54 | 43.57 | 46.36 |
| *LjbZIP28* | 0.44 | 0.3 | 0.38 | 0.24 | 0.15 | 0.3 | 0.28 | 0.32 | 0.29 | 1.62 | 1.52 | 1.25 | 0.88 | 1.03 | 1.08 |
| *LjbZIP29* | 56.69 | 63.93 | 65.21 | 56.69 | 84.07 | 55.54 | 48.58 | 51.82 | 45.86 | 13.95 | 11 | 19.9 | 23.91 | 19.58 | 20.06 |
| *LjbZIP30* | 22.2 | 21.61 | 25.35 | 32.09 | 39.49 | 31.82 | 28.1 | 24.16 | 24.58 | 41.38 | 39.49 | 38.04 | 31.73 | 31.74 | 29.56 |
| *LjbZIP31* | 0.9 | 0.81 | 0.33 | 1.55 | 1.89 | 1.3 | 31.6 | 26.02 | 33.39 | 26.85 | 18.19 | 27.86 | 0.75 | 0.52 | 0.51 |
| *LjbZIP32* | 1.66 | 1.5 | 1.34 | 1.78 | 2.74 | 2.2 | 0.98 | 1.36 | 1.13 | 0 | 0 | 0 | 0 | 0 | 0 |
| *LjbZIP33* | 6.37 | 4.87 | 5.38 | 2.62 | 1.91 | 2.78 | 9.16 | 6.09 | 8.24 | 17.08 | 14.08 | 13.94 | 6.46 | 6.95 | 7.12 |
| *LjbZIP34* | 2.06 | 1.7 | 1.83 | 3.49 | 1.48 | 1.61 | 1.26 | 2.35 | 2.89 | 1.91 | 2.2 | 2.71 | 1.69 | 1.73 | 2.61 |
| *LjbZIP35* | 1.56 | 1.56 | 1.21 | 1.16 | 1.5 | 1.05 | 0.4 | 1.04 | 0.34 | 10.37 | 10.29 | 12.4 | 32.47 | 32.02 | 34.15 |
| *LjbZIP36* | 5.94 | 5.73 | 5.9 | 3.57 | 4.38 | 3.53 | 1.31 | 3.29 | 1.29 | 3.83 | 3.24 | 4.39 | 8.61 | 8.85 | 10.52 |
| *LjbZIP37* | 33.55 | 32.99 | 34.51 | 12.19 | 28.38 | 12.96 | 3.93 | 20.92 | 2.86 | 0.38 | 0.34 | 0.51 | 0.67 | 0.47 | 0.58 |
| *LjbZIP38* | 17.9 | 15.44 | 20.26 | 24.01 | 25.75 | 23.62 | 21.48 | 22.05 | 20.12 | 13.45 | 12.05 | 12.22 | 17.03 | 16.55 | 18.69 |
| *LjbZIP39* | 23.82 | 24.16 | 19.53 | 20.22 | 17.98 | 19.09 | 18.06 | 21.71 | 21.83 | 18.93 | 18.41 | 14.9 | 11.05 | 12.02 | 8.98 |
| *LjbZIP40* | 284.57 | 298.73 | 300.98 | 305.35 | 330.85 | 299.22 | 225.6 | 270.24 | 212.48 | 34.32 | 31.88 | 38.16 | 19.4 | 23.49 | 23.28 |
| *LjbZIP41* | 27.09 | 27.55 | 26.72 | 28.31 | 36.35 | 28.94 | 27.3 | 27.95 | 23.43 | 34.99 | 32.5 | 37.67 | 36.01 | 35.81 | 36.36 |
| *LjbZIP42* | 0.06 | 0.21 | 0.12 | 0.3 | 0.31 | 0.31 | 0.48 | 0.13 | 0.38 | 0.78 | 1.67 | 1.26 | 0.78 | 0.41 | 0.34 |
| *LjbZIP43* | 34.14 | 43.3 | 41.17 | 40.03 | 52.37 | 36.84 | 48.54 | 49.76 | 44.95 | 46.18 | 39.9 | 67.52 | 74.59 | 81.25 | 74.17 |
| *LjbZIP44* | 9.64 | 8.6 | 9.57 | 8.58 | 8.91 | 9.73 | 21.47 | 14.42 | 19.81 | 28.72 | 27.36 | 25.84 | 19.14 | 19.44 | 21.3 |
| *LjbZIP45* | 65.63 | 72.6 | 80.77 | 45.79 | 42.8 | 48.73 | 70.95 | 73.3 | 75.54 | 22.52 | 22.13 | 25.11 | 24.97 | 30.05 | 26.72 |
| *LjbZIP46* | 4.45 | 3.74 | 4.71 | 3.4 | 2.91 | 2.74 | 0.86 | 1.84 | 0.92 | 0.34 | 0.4 | 0.49 | 0.51 | 0.68 | 0.59 |
| *LjbZIP47* | 7.28 | 5.75 | 6.43 | 4.2 | 3.37 | 3.61 | 2.08 | 3.05 | 2.09 | 1.68 | 1.11 | 1.43 | 0.22 | 0.19 | 0.28 |
| *LjbZIP48* | 10.21 | 10.65 | 9.53 | 11.38 | 10.58 | 9.94 | 18.97 | 13.27 | 17.15 | 6.07 | 3.84 | 5.53 | 2.58 | 2.61 | 2.46 |
| *LjbZIP49* | 0.57 | 0.37 | 0.97 | 0.35 | 0.29 | 0.29 | 0.33 | 0.46 | 0.53 | 1 | 0.65 | 1.84 | 1.95 | 0.95 | 1.3 |
| *LjbZIP50* | 32.69 | 32.02 | 30 | 26.27 | 17.74 | 23.92 | 16.85 | 18.92 | 18.81 | 27.88 | 27.11 | 25.05 | 25.28 | 24.92 | 20.37 |
| *LjbZIP51* | 28.45 | 27.01 | 29.71 | 26.81 | 20.26 | 30.63 | 26.79 | 27.84 | 30.55 | 32.49 | 32.06 | 24.88 | 21.56 | 24.95 | 23.34 |
| *LjbZIP52* | 33.42 | 30.57 | 30.97 | 37.42 | 38.41 | 36.68 | 35.29 | 33.36 | 34.08 | 32.57 | 30.98 | 30.02 | 15.42 | 14.59 | 13.64 |
| *LjbZIP53* | 99.09 | 99.03 | 94.97 | 87.1 | 84.92 | 89.46 | 184.51 | 159 | 172.91 | 282.94 | 266.23 | 286.76 | 193.88 | 206.18 | 198.76 |
| *LjbZIP54* | 72.19 | 82.74 | 80.14 | 100.48 | 99.06 | 101.71 | 198.54 | 128.18 | 185.09 | 45.86 | 45.51 | 42.31 | 15.67 | 15.87 | 16.3 |
| *LjbZIP55* | 1.82 | 2.31 | 1.81 | 4.67 | 4.88 | 5.21 | 3.13 | 2.07 | 2.83 | 1.31 | 0.79 | 1.75 | 0.14 | 0.31 | 0 |
| *LjbZIP56* | 8.07 | 7.62 | 7.64 | 9.83 | 9.12 | 10.85 | 8.64 | 7.84 | 7.78 | 0.33 | 0.6 | 0.44 | 0.13 | 0.2 | 0.17 |
| *LjbZIP57* | 3.27 | 2.21 | 2.53 | 2.55 | 2.97 | 2.28 | 3.45 | 4.59 | 3.12 | 6.34 | 5.25 | 6.59 | 2.56 | 3.23 | 3.13 |
| *LjbZIP58* | 35.76 | 35.55 | 37.05 | 33.3 | 30.99 | 36.2 | 34.08 | 34.21 | 31.92 | 33.21 | 30.63 | 24.6 | 27.2 | 26.25 | 29.32 |
| *LjbZIP59* | 4.85 | 7.24 | 8.09 | 6.46 | 9.88 | 7.97 | 7.57 | 8.63 | 10.4 | 29.5 | 31.84 | 65.08 | 53.9 | 54.8 | 51.24 |
| *LjbZIP60* | 44.87 | 46.33 | 42.52 | 47.24 | 28.13 | 43.41 | 40.86 | 34.23 | 38.55 | 30.55 | 31.62 | 25.67 | 21.38 | 23.84 | 24.51 |
| *LjbZIP61* | 42.52 | 43.25 | 42.3 | 92.77 | 105.84 | 93.03 | 38.19 | 38.33 | 37.27 | 30.26 | 26.23 | 27.85 | 29.12 | 29.47 | 30 |
| *LjbZIP62* | 12.27 | 14.26 | 10.44 | 7.56 | 9.31 | 9.06 | 7.98 | 11.64 | 10.36 | 12.63 | 12.05 | 11.86 | 13.41 | 14.98 | 12.53 |
| *LjbZIP63* | 8.35 | 8.29 | 8 | 7.22 | 11.15 | 7.97 | 9.9 | 7.72 | 8.93 | 10.81 | 10.83 | 11.23 | 10.81 | 8.78 | 12.12 |
| *LjbZIP64* | 37.79 | 35.53 | 38.02 | 37.43 | 26.01 | 37.94 | 38.79 | 34.97 | 36.33 | 54.06 | 52.79 | 46.49 | 47.14 | 47.22 | 46.63 |
| *LjbZIP65* | 0 | 0 | 0 | 0 | 0 | 0 | 0 | 0 | 0 | 0 | 0 | 0 | 0 | 0 | 0 |
| *LjbZIP66* | 1.75 | 2.17 | 4.36 | 2.16 | 1.13 | 2.31 | 1.71 | 1.95 | 1.7 | 4.81 | 2.95 | 2.65 | 2.27 | 3.16 | 2.16 |
| *LjbZIP67* | 0 | 0 | 0 | 0 | 0 | 0 | 0 | 0 | 0 | 0 | 0 | 0 | 0 | 0 | 0 |
| *LjbZIP68* | 5.53 | 8.38 | 10.5 | 7.88 | 9.36 | 8.14 | 8.17 | 10.07 | 8.02 | 24.92 | 34.17 | 63.25 | 46.68 | 47.47 | 47.91 |

**Table S6** Comprehensive screening information of *LjbZIP* family members associated with β-carotene accumulation during floral development (Pearson's |r| ≥ 0.8, p-value ＜ 0.05 ).

| Gene name | Gene ID | Peak Stage | Compounds | coefficient | p-value | Phylogenetic subgroup | Prioritized |
| --- | --- | --- | --- | --- | --- | --- | --- |
| *LjbZIP17* | IMPGLJA1N29867 | S5 | β-carotene | 0.86 | 3.89e-5 | Group S | No |
| *LjbZIP19* | IMPGLJA1N24514 | S1 | β-carotene | -0.90 | 5.32e-6 | Group D | No |
| *LjbZIP35* | IMPGLJA1N99277 | S5 | β-carotene | 0.90 | 3.67e-6 | Group D | No |
| *LjbZIP52* | IMPGLJA1N83378 | S2 | β-carotene | -0.93 | 6.46e-7 | Group G | No |
| *LjbZIP36* | IMPGLJA1N98341 | S5 | β-carotene | 0.90 | 6.54e-6 | Group A | Yes |

**Table S7** Data of quantitative real-time PCR.

|  | Repeat1 | Repeat2 | Repeat3 | Mean | SD | Gene |
| --- | --- | --- | --- | --- | --- | --- |
| EV | 0.821690315 | 0.997692177 | 1.21981864 | 1.013067044 | 0.199508974 | *NbPSY* |
| *LjbZIP36-OE* | 1.522736872 | 1.67791155 | 1.74916534 | 1.649937921 | 0.115777181 |  |
| EV | 1.130269389 | 0.950439478 | 0.930879716 | 1.003862861 | 0.109907251 | *NbPDS* |
| *LjbZIP36-OE* | 1.420763739 | 1.38191288 | 1.465778051 | 1.422818224 | 0.041970316 |  |
| EV | 0.821690315 | 1.01865581 | 1.194715135 | 1.011687087 | 0.186610025 | *NbZDS* |
| *LjbZIP36-OE* | 1.38191288 | 1.420763739 | 1.450617005 | 1.417764542 | 0.034450118 |  |
| EV | 0.926588062 | 1.101905116 | 0.979420298 | 1.002637825 | 0.089935018 | *NbLCYB* |
| *LjbZIP36-OE* | 1.450617005 | 1.470866864 | 1.554732811 | 1.492072227 | 0.055202136 |  |

**Table S8** Method validation parameters for carotenoid quantification by LC-MS/MS.

| Analytes | Linear Range | R^2^ | LLOQ (Lower limit of quantification) | ULOQ (Upper limit of quantification) | Intra-day precision | Inter-day precision | Recovery (%) | External standard method/ Internal standard method |
| --- | --- | --- | --- | --- | --- | --- | --- | --- |
|  | (μg/mL) | (μg/mL) | (μg/mL) | (μg/mL) | (RSD% N=3) 5μg/mL | (RSD% N=3) 5μg/mL |  |  |
| β-carotene | 0.0025-25 | 0.9948 | 0.0025 | 25 | 1.50 | 9.63 | 109.11 | External standard method |
| (E/Z)-phytoene | 0.05-25 | 0.99933 | 0.05 | 25 | 0.61 | 16.08 | 100.27 | External standard method |
